# Sex-dependent immune activation shapes disease progression in a model of Parkinson’s disease

**DOI:** 10.1186/s13293-025-00809-1

**Published:** 2025-12-24

**Authors:** Leah C. Beauchamp, Lily A. Palumbo, Toby B. Lanser, Mariam Baig, Laura M. Cox

**Affiliations:** Ann Romney Center for Neurologic Diseases, Brigham & Women’s Hospital, Harvard Medical School, 60 Fenwood Road, Boston, MA USA

**Keywords:** Parkinson’s disease, Microglia, T cells, Sex, Aging, Cognition, 3KL, Alpha synuclein

## Abstract

**Background:**

While it is clear that inflammation contributes to Parkinson’s disease (PD) and prevalence is higher in males, sex remains an underexplored determinant of immune responses in PD.

**Methods:**

Using the 3KL transgenic mouse model, which expresses three E to K α-synuclein mutations, we investigated how sex and age shape peripheral and central immunity and behavior in synucleinopathy. Male and female 3KL mice were aged to 8- and 14-months. At these ages animals underwent motor and cognitive assessment, followed by assessment of the peripheral immune response using flow cytometry and analysis of microglial transcriptional profiles by bulk RNA sequencing.

**Results:**

Male 3KL mice exhibited earlier onset and greater severity of motor and cognitive impairments, which was linked to a pro-inflammatory peripheral immune profile marked by increased cytotoxic CD8⁺ T cells and IFNγ-producing CD4 Th1 cells. In contrast, female mice displayed delayed symptom onset, preserved cognition, along with early elevations in regulatory IL-10⁺ CD4 and γδ T cells. RNA sequencing of microglia revealed broad sex differences at 8 months. Males demonstrated early upregulation of microglia neurodegenerative signatures, MHC class I/II signaling, ceramide signaling, and pronounced lipid dysregulation, while females showed upregulation of microglial pathways related to protein, metabolic, and neuronal maintenance, including phagosome formation, docosahexaenoic acid signaling, and synaptogenesis pathways. Microglial transcriptional differences were nearly absent by 14 months, suggesting sex-specific trajectories converge during late-stage disease, which is concurrent with a decrease in estrogen in aged female mice.

**Conclusions:**

Together, these findings reveal distinct immune signaling in male and female 3KL mice and identify coordinated changes in T cell and microglial responses that may contribute to sex differences in PD vulnerability and progression. This work underscores the importance of incorporating sex as a biological variable in neurodegeneration research and provides mechanistic insight into immune-mediated modulation of synucleinopathy.

**Supplementary Information:**

The online version contains supplementary material available at 10.1186/s13293-025-00809-1.

## Abstract (plain language summary)

Parkinson’s disease (PD) affects millions of people worldwide and is more common in men than women. Scientists believe that differences in the immune system may help explain this, but little is known about how sex and age shape these responses during the disease. In this study, we used a mouse model of PD that develops symptoms similar to those seen in people, including problems with movement and memory. We compared male and female mice at two ages: middle age (8 months) and older age (14 months). We looked at both behavior and how the immune system in the body and brain responded. Male mice showed earlier and more severe movement and memory problems. Their immune systems were more inflammatory, with high levels of aggressive immune cells. Female mice were protected for longer, performing better on memory tests, and had more “regulatory” immune cells that help keep inflammation in check. When we studied microglia, the immune cells of the brain, we found striking sex differences. In males, microglia turned on genes linked to nerve cell damage and inflammation. In females, microglia activated genes that support brain health. These differences were strongest at 8 months but faded with age, coinciding with the natural drop in estrogen in older females. Overall, our results show that sex strongly shapes the immune response in PD. Understanding these differences may lead to more personalized approaches to treatment and prevention.

**Supplementary Information:**

The online version contains supplementary material available at 10.1186/s13293-025-00809-1.

## Highlights

- Male 3KL mice exhibited earlier and more severe motor and cognitive decline, linked to a pro-inflammatory peripheral immune profile.
- Female mice showed delayed symptom onset and preserved cognition, associated with early increases in regulatory IL-10⁺ CD4 and γδ T cells.
- Microglial RNA sequencing revealed broad sex differences at 8 months: males upregulated neurodegenerative and lipid dysregulation pathways, while females upregulated protein maintenance, phagosome formation, DHA signaling, and synaptogenesis.
- These sex-specific microglial transcriptional programs diminished by 14 months, coinciding with decreased estrogen in aged females.
- Findings identify coordinated differences in T cell and microglial responses that may underlie sex-specific vulnerability and resilience in Parkinson’s disease.

**Supplementary Information:**

The online version contains supplementary material available at 10.1186/s13293-025-00809-1.

## Background

Parkinson’s disease (PD) is a prevalent neurodegenerative disorder, impacting around 1% of individuals over the age of 60 [1]. While recent studies suggest the male-to-female ratio has narrowed from ~ 1.5:1 [2] to ~ 1.2:1 [3], sex remains a critical determinant of PD risk, with differences in incidence most apparent between ages of 50–59 [4]. Age of onset is later in women than men [5], and women with PD initially present with a more benign phenotype [6]. Despite this, women have a faster progression of disease and higher mortality rate [7]. Sex differences in incidence, diagnosis and progression also exist in other -synucleinopathies, including dementia with Lewy bodies (DLB) and multiple systems atrophy (MSA) [8]. Several factors may contribute to these disparities, including sex-associated environmental exposures, such as increased risk from historically male-dominated occupations, and hormonal differences, as estrogen may exert neuroprotective effects that delay or reduce the risk of PD in females [9–11]. Moreover, sex-specific immune responses to pathological processes and aging are highly divergent and may play a key role in modulating disease susceptibility.

Aggregated α-synuclein acts as a pro-inflammatory stimulus that disrupts both central and peripheral immune homeostasis [12, 13]. Peripheral T cells recognize α-synuclein peptides [14], which is associated with early PD [15]. In the brain, extracellular aggregates activate microglia [16, 17], leading to the release of pro-inflammatory cytokines and promoting a chronic neuroinflammatory state that contributes to neuronal damage [17]. These aggregates can also impair microglial phagocytic function, reducing their ability to clear pathological proteins [18]. Importantly, microglial response to -synuclein is thought to be conformation-specific; wild-type and A53T α-synuclein enhance phagocytosis, whereas the E46K -synuclein variants inhibit it [19].

The E46K mutation in *SNCA* is observed in cases of PD, PD with dementia (PDD), and DLB. This mutation causes an autosomal dominant synucleinopathy characterized by early-onset parkinsonism, rapid progression, and prominent dementia with psychiatric and autonomic features [20]. Pathologically, it is associated with widespread cortical and limbic Lewy body pathology, overlapping PD and DLB [20]. This mutation is modelled in the 3KL α‑synuclein transgenic mouse model along with two additional E to K mutations [21]. The 3KL mice present with consistent sex differences in which males exhibit earlier onset and more severe motor deficits including impaired pole descent and poorer rotarod performance starting as early as ~ 10 weeks and becoming pronounced by 6 months. Females, by contrast, display delayed and milder symptoms across all these behavioral assays, with pronounced motor symptoms seen at 6 months, albeit milder than males [22]. On a molecular level, male 3KL mice accumulate more pathological α‑synuclein monomers and phosphorylated Ser129 aggregates in cortex and hippocampus, whereas females maintain higher tetramer:monomer ratios and greater soluble ‑synuclein, correlating with less synaptic pathology and delayed cognitive impairment [23].

Understanding sex differences in PD is essential for uncovering distinct or divergent pathological processes in males and females, with the ultimate goal of enabling more precise and effective therapeutic strategies. The 3KL transgenic mouse model, which harbors three α-synuclein mutations and exhibits pronounced sexual dimorphism in motor and pathological outcomes, offers a unique opportunity to investigate how synucleinopathies differentially shape the immune landscape across sexes. Despite increasing recognition of the role of neuroinflammation in PD, no study has comprehensively examined how peripheral and central immune responses, specifically T cell and microglial populations, interact with sex and age in this model.

Here, we test the hypothesis that sex-dependent differences in behavioral outcomes in 3KL mice are associated with distinct peripheral immune profiles and microglial activation states. Specifically, we predict that male mice will show earlier pro-inflammatory immune changes coinciding with greater motor and cognitive decline, whereas female mice will display delayed or more regulatory immune responses that parallel relative preservation of function. By characterizing these age- and sex-dependent immune alterations, this study aims to provide new insight into the immunopathology of synucleinopathies.

## Methods

### Mice

The 3KL mice are a transgenic model that express human α-synuclein with three E→K mutations expressed under the Thy1.2 promoter, including the E46K mutation, associated with familial PD [21]. Mice were generated at our center by Nuber *et al*. as previously described (Line #3798, Jax # 032799) [21] and bred to homozygosity. Female and male 3KL α-synuclein mice were bred in-house and maintained under standard specific-pathogen free conditions under at 12-hour light/dark cycle with access to food and water *ad libitum*. Mice were aged to either 8- or 14-months in grouped housing conditions (max 5 mice per cage). For this study, *n* = 7 female and male at 8 months and *n* = 6 female and male at 14 months were used. We used 6–7 mice per group, which was determined based on sample size calculations from our bulk RNA-seq experiments. This group size provided sufficient power to detect transcriptomic differences of interest while remaining consistent with prior studies in the 3KL model. All animal procedures were approved by the Institutional Animal Care and Use Committee at Brigham & Women’s Hospital (IACUC protocol #2023N000080). Following behavioral analyses, mice were euthanized with CO_2_ until respiratory arrest. Blood was extracted from the heart and animals were perfused by injecting 30 ml of cold Hank’s buffered saline solution (HBSS) buffer into the left ventricle of the heart. Following perfusion, the brain, spleen, and cervical lymph nodes were collected.

### Behavioral analysis

**Fig. 1:**
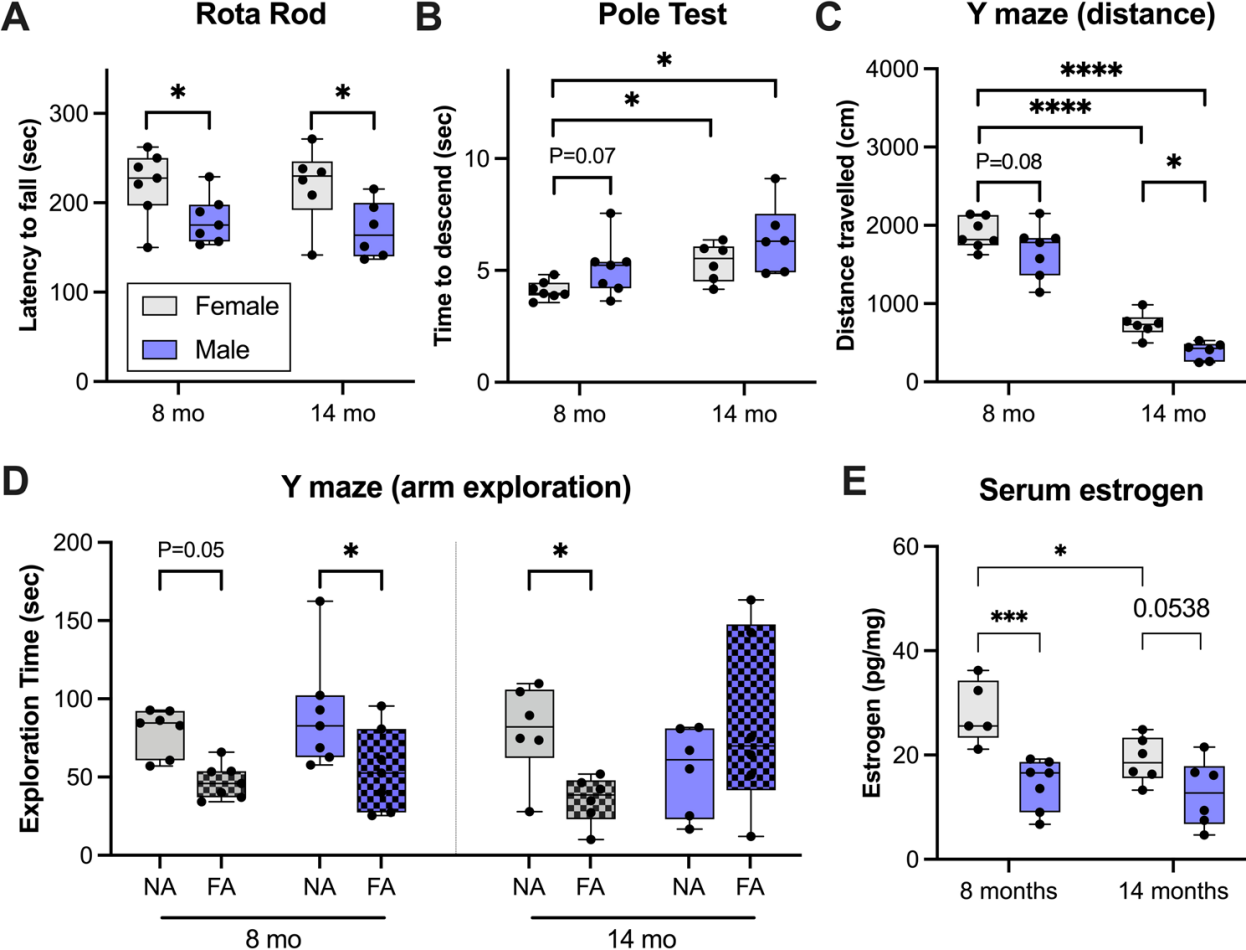
Male 3KL mice display accelerated motor and cognitive decline compared to females.** (a)** Latency to fall on the accelerating rotarod, **(b)** time to complete the pole test, **(c)** distance travelled in the blocked arm Y-maze, and **(d)** duration of time spent exploring the novel arm (NA) and familiar arm (FA) in the blocked arm Y maze. **(e)** Mouse serum estrogen levels (pg/mg protein). *N* = 6–7 per group. Results are presented as a box and whisker plot, with whiskers representing minimum and maximum values. Rota rod, pole test, distance travelled on the Y maze and estrogen levels were analyzed using linear mixed-effects models with sex, age, and their interaction as fixed effects. Post hoc comparisons were performed using uncorrected Fisher’s Least Significant Difference test following significant main or interaction effects. Novel arm exploration on the Y maze was compared to an average of time spent exploring the two previously explored arms (familiar arms) and was analyzed using a two-way ANOVA with Tukey’s multiple comparison test. *P* < 0.05 was considered significant. *P < 0.05, ****P < 0.0001. F: female, FA: familiar arms, M: male, NA: novel arm. Overall, these findings indicate that male 3KL mice develop earlier and more severe motor impairments and age-related cognitive decline, whereas females are relatively protected until later in life, in part aligning with differences in estrogen levels

For all behavioral assays, mice were habituated in the testing room for 60 min without human interaction, with cage filters removed to allow free exchange of room air and scents. To minimize pheromonal influence, males were always tested before females, and sexes were never present in the room simultaneously. Between trials, apparatuses and boxes were cleaned with Peroxigard. Details of the statistical methods for each behavioral measure are provided in the Fig. 1 legend.

*Rota Rod*: Gross motor coordination was evaluated using a rotarod apparatus (Ugo Basile). On Day 1, mice were habituated to the stationary rod for 30 s, followed by three habituation trials spaced 30 min apart. The first two habituation trials were conducted at a constant speed of 4 rpm for 120 s each. In the third habituation trial, the rod was set to accelerate from 4 to 40 rpm over a 5-minute period, although mice remained on the rod for only 2 min to minimize fatigue. On Day 2, motor performance was assessed by measuring latency to fall on an accelerating rod (4–40 rpm over 5 min). Each mouse underwent three 5-minute trials, with a one-hour inter-trial interval, and the average time spent on the rod before falling was recorded as a measure of motor coordination and endurance.

*Pole Test*: Motor coordination was evaluated using the vertical pole test. Mice were trained the day before testing to acclimate to the pole. Mice were placed on a metal pole wrapped with gauze (50 cm high, 1 cm diameter, placed vertically), with their head oriented towards the top of the pole. Mice were required to complete three turn and descent maneuvers on the training day, with a maximum time to complete of 120 s, though no mice exceeded this time. 24 h later, mice performed three trials which were recorded to track the turn time, descent time, and total time. The three trials were averaged to generate a composite score for each mouse.

*Y-maze*: Spatial recognition memory was assessed by exposing mice to a two-trial Y-maze task with a 1-hour intertrial interval. Three identical arms of the maze (arms: 35 cm length x 5 width x 10 height; radially arranged) were randomly assigned a visual cue at the end of arm and one arm was designated the novel arm. In the first trial (acquisition), mice were placed in the maze and allowed to explore all but the blocked off novel arm for 5 min. For the second trial (retention), mice were allowed to explore the entire maze for 5 min. Duration in arms and distance travelled was recorded by an overhead video camera and analyzed using the ANY-Maze software. The two previously explored arms (start arm and familiar arm) were averaged and compared to time exploring the novel arm.

### Estrogen levels

Upon euthanasia, animals were exsanguinated via cardiac puncture. Whole blood was collected and allowed to coagulate at room temperature for 1 h. Whole blood was centrifuged at 2000 rpm for 10 min at room temperature to separate the serum fraction, which was stored at −80 °C. Serum estrogen levels were measured using a mouse estrogen ELISA kit (ab285291, Abcam) per manufacturer’s instructions. Briefly, serum samples were incubated with Biotin-detection anti-estrogen antibody, followed by HRP-Streptavidin Conjugate and TMB substrate with washes and incubation conditions as recommended. The reaction was stopped and absorbance measured at 450 nm immediately on a GloMax Explorer plate reader (Promega). Samples were normalized to serum protein concentration as determined by BCA (ThermoScientific).

### Preparation and stimulation of peripheral immune cells for flow cytometry

Spleens and cervical lymph nodes were isolated and mechanically dissociated through a 70 μm filter in sterile phosphate-buffered saline. Red blood cells in spleen samples were lysed using ammonium-chloride-potassium lysis buffer (ACK). Single-cell suspensions were resuspended in complete Iscove’s Modified Dulbecco’s Medium supplemented with 10% fetal bovine serum, 1% penicillin-streptomycin, 0.1% β-mercaptoethanol, and 1% non-essential amino acids. To induce cytokine expression, cells were stimulated with phorbol 12-myristate 13-acetate (PMA, 50 ng/ml), ionomycin (1 µM), and GolgiStop (1 µg/ml; BD Biosciences) for 3 h at 37 °C in 5% CO₂.

Following PMA/ionomycin stimulation, cells were washed and incubated with anti-CD16/CD32 (Fc Block; BD Biosciences) for 10 min. Zombie Aqua Fixable Viability Dye (BioLegend, 1:1000 in PBS) was used to exclude dead cells. Surface staining was performed at 4 °C for 20 min using fluorochrome-conjugated antibodies listed in Supplementary Tables 1 and 2. Cells were then fixed and permeabilized using the Foxp3/Transcription Factor Staining Buffer Set (eBioscience) according to the manufacturer’s instructions. Intracellular staining was performed in permeabilization buffer at room temperature for 30 min. After a final wash, samples were resuspended in FACS buffer (10% fetal bovine serum, 2.5% 4- (2-hydroxyethyl)−1-piperazineethanesulfonic acid (HEPES) and 0.4% ethylenediaminetetraacetic acid (EDTA) in HBSS). Samples were analyzed on a BD FACSymphony™ flow cytometer (BD Biosciences) and analyzed using FlowJo software (BD Biosciences). Differences in immune populations by age and sex were determined by mixed effect analysis with post-hoc Fisher’s LSD in GraphPad Prism.

### Microglia isolation and bulk RNA sequencing

The right hemi-brain was homogenized using a 7 mL Dounce glass homogenizer, and immune cells were isolated by 37% Percoll (GE Healthcare) centrifugation. Immune cells were incubated with CD45-FITC, CD11b-PE-Cy7, ASCA2-PE and a combination of lineage negative antibodies tagged to the fluorophore PerCP (Supplementary Table 3) for 20 min at room temperature. Cells were washed with FACS buffer, filtered through a 35 μm cell strainer and added to 5 µl 7-AAD live-dead stain (BD Biosciences). Microglia were identified as CD45^int^ CD11b^+^ Ly6c^−^. 2000 microglia were sorted using a BD FACSAria II cell sorter and dry pellets were stored at −80 °C.

2000 microglial cells were thawed on ice and lysed in 10 µl TCL buffer (Qiagen) with 1% beta-mercaptoethanol (Gibco) and 5 µl (1000 cells) were loaded onto plates in preparation for sequencing. cDNA libraries were performed in accordance with the Smart-seq2 protocol [24] by the Broad Technology Labs and sequenced by the Broad Genomics Platform. Illumina NextSeq500 and the High Output v2 kit were used to generate 2 × 25 bp reads. Transcripts were quantified using the BTL computational pipeline with Cuffquant (v2.2.1). Raw counts were normalized using the median-of-ratios method implemented in DESeq2 (version 1.36.0) [25]. Genes with an average read count below 100 were excluded to prioritize those with greater relevance to microglial biology. Transcriptome alignments were conducted using the Salmon tool with its standard parameters [26]. The R software (version 4.2.1) and DESeq2 package were employed to analyze differential gene expression. Differential expression analysis was conducted using DESeq2’s Wald test applied to relevant variable contrasts. P-values were adjusted for multiple comparisons using the Benjamini–Hochberg procedure. Pathway analysis was conducted using Ingenuity Pathway Analysis (QIAGEN) for genes with a fold change > ± 1 and unadjusted p-value < 0.05.

## Results

### Male 3KL mice exhibit earlier onset of motor and cognitive deficits compared to females

3KL male mice show an accelerated motor and cognitive phenotype, consistent with females being partially protected by estrogen [22, 23]. In this study, motor coordination and endurance was assessed using the rotarod at 8 and 14 months of age (Fig. 1a**)** and showed a significant main effect of sex (*P* = 0.01), with male performance significantly reduced vs. females at both 8 and 14 months of age (*P* = 0.04 and *P* = 0.02, respectively). Motor coordination and bradykinesia were evaluated using the pole test (Fig. 1b**)**. There were significant main effects of sex (*P* = 0.048) and age (*P* = 0.008). At 8 months, male mice trended toward slower performance vs. females (*P* = 0.07). From 8 to 14 months, both sexes showed a significant age-related decline in performance (female: *P* = 0.047; male: *P* = 0.04), but there were no differences between 14-month males and females on the pole test.

Exploratory behavior and spatial working memory were tested using the blocked arm Y-maze. Exploratory activity showed significant main effects of sex (*P* = 0.004) and age (*P* < 0.0001) (Fig. 1c**)**. While male mice showed a non-significant reduction in exploration at 8 months vs. female mice (*P* = 0.08), this became significant by 14 months (*P* = 0.02). Both sexes demonstrated a significant decline in exploration from 8 to 14 months (*P* < 0.0001). Spatial working memory remained intact in both sexes at 8 months as determined by increased novel arm exploration (female: *P* = 0.051; male: *P* = 0.03) Fig. 1d**)**. At 14 months, female mice retained novel arm preference (*P* = 0.01), whereas male mice no longer displayed this preference, indicating that male but not female 3KL mice demonstrate age-related cognitive impairment.

To determine whether sex-specific changes in motor and cognitive decline were linked to age-related alterations in circulating estrogen, we measured estrogen levels, as previous findings suggested that estrogen may confer protection in 3KL female mice [22]. At 8 months of age, when females are not behaviorally impaired, there was a significant 2-fold higher level of circulating estrogen in female mice compared with males (*P* = 0.0003). Estrogen levels significantly decreased in female mice from 8 to 14 months (*P* = 0.01) and by 14 months when the females have succumbed to motor impairment, there is only a trend (*P* = 0.053) of higher estrogen than males **(**Fig. 1e**)**.

To help contextualize behavioral performance relative to age-matched WT mice, we include average values from 8- and 14-month-old male and female WT animals in Supplementary Table 4. These animals were not part of the current experiment and should not be interpreted as experimental controls; they are provided solely to aid interpretation of the results.

## Sex- and age-driven shifts in effector and regulatory T cell populations in 3KL mice

Because age-related changes in inflammation can contribute to PD pathogenesis, we assessed peripheral immune alterations in the cervical lymph node (CLN) and spleen of male and female 3KL mice at 8 and 14 months of age. We found main effects of both sex and age across our various immunologic phenotypes (statistical factors reported in Supplementary Table 5).

**Fig. 2:**
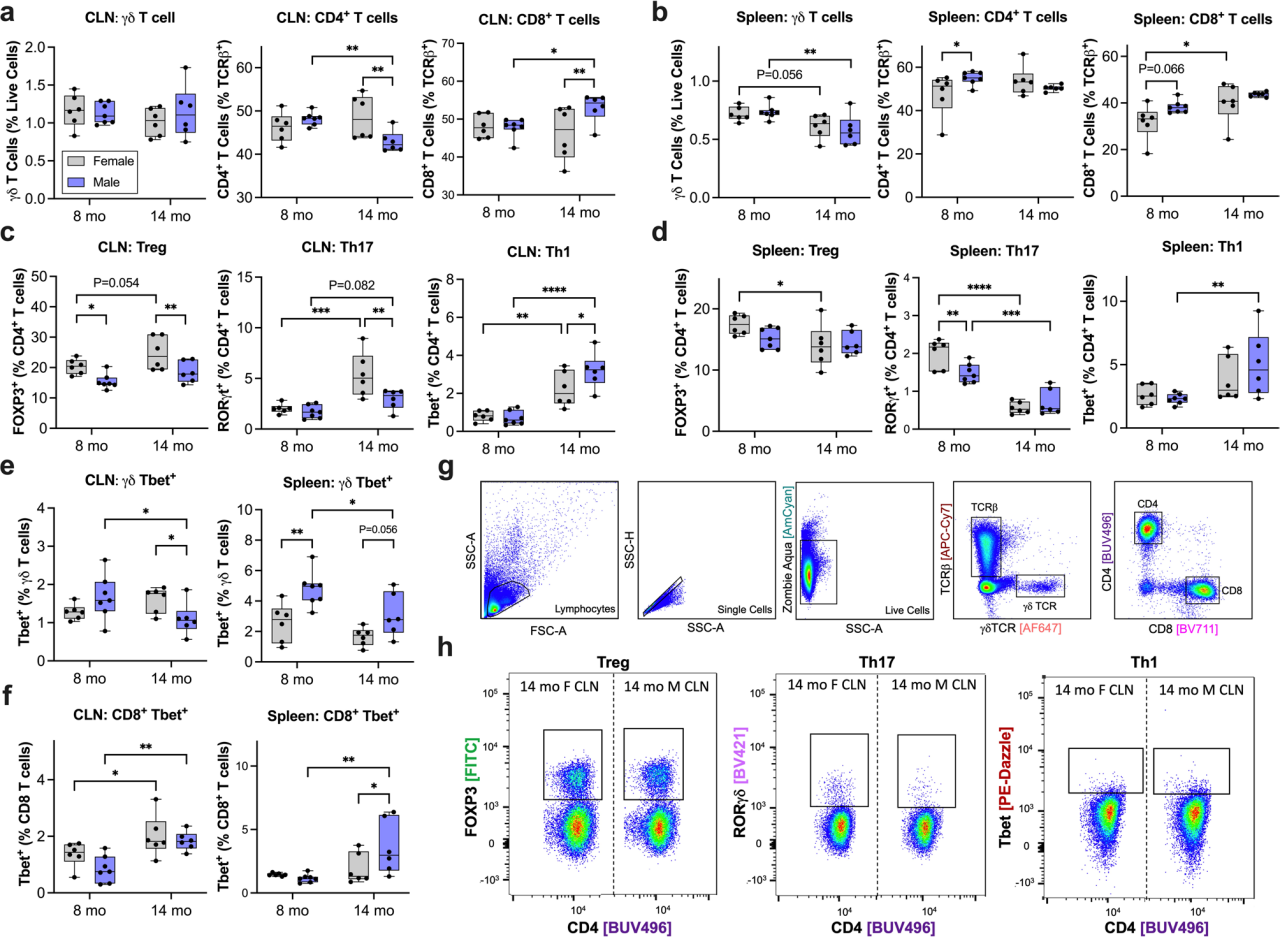
Age and sex-dependent T cell alterations in 3KL mice.** (a)** γδ, CD4^+^, and CD8^+^ T cell sub-sets in the cervical lymph nodes. **(b)** γδ, CD4^+^, and CD8^+^ T cell sub-sets in the spleen. **(c)** CD4^+^ T cell sub-types of Tregs, Th17, and Th1 in the cervical lymph nodes. **(d)** CD4^+^ T cell sub-types of Tregs, Th17, and Th1 in the spleen. **(e)** Tbet^+^ γδ T cells from cervical lymph node and spleen. **(f)** Tbet^+^ CD8^+^ T cells from cervical lymph node and spleen. **(g)** Gating strategy to isolate γδ, CD4^+^, and CD8^+^ T cells. **(h)** Representative pseudocolor plots from 14 mo CLN samples for females (left) and males (right) of Treg, Th17, and Th1 T cells. Whiskers of box and whisker plot represent minimum and maximum. Grey boxes are female data; blue boxes are male data. Data were analyzed by mixed effect analysis with post-hoc Fisher’s LSD. *P* < 0.05 was considered significant. *P < 0.05, **P < 0.01, ***P < 0.001, ***P < 0.0001

Examining early sex-specific differences at 8 months, male 3KL mice had increased splenic CD4^+^ T cells (*P* = 0.04) and Tbet^+^ γδ T cells (*P* = 0.006) **(**Fig. 2b and e**)**, and reduced FoxP3^+^CD4^+^ Tregs in the CLN (*P* = 0.02) and splenic RORγT^+^CD4^+^ Th17 cells (*P* = 0.004) **(**Fig. 2c and d**)**. By 14 months of age at late-stage disease, males had increased CD8^+^ T cells (*P* = 0.006) and Tbet^+^ Th1 cells in the CLN (*P* = 0.03) **(**Fig. 2a and c**)** and increased Tbet^+^ γδ (*P* = 0.052) and CD8^+^ T cells (*P* = 0.03) in the spleen **(**Fig. 2e and f**)**. Conversely, males have reduced CD4^+^ (*P* = 0.004), Treg (*P* = 0.009), Th1 (*P* = 0.003), and Tbet^+^ γδ T cells (*P* = 0.0498) in the CLN compared to females at 14 months of age **(**Fig. 2a, c and e**)**.

We also examined age-related immune changes separately in females and males. 14-month-old females had reduced splenic γδ T cells (*P* = 0.056), Tregs (*P* = 0.02) and Th17 cells (*P* < 0.0001) **(**Fig. 2b and d**)**, a trend to increased splenic CD8^+^ T cells (*P* = 0.07) and CLN Tregs (*P* = 0.054) **(**Fig. 2b and c**)**, and significantly increased CLN Th17 (*P* = 0.001), Th1 (*P* = 0.002), and Tbet^+^ CD8^+^ T cells (*P* = 0.04) **(**Fig. 2c and f**)**. At 14 months, males had reduced CD4^+^ and Tbet^+^ γδ T cells in the CLN **(**Fig. 2a and e**)** and γδ T cells (*P* = 0.005), Th17 (*P* = 0.0001) and Tbet^+^ T cells (*P* = 0.005) in the spleen **(**Fig. 2b and d, and 2e**)** compared to 8 months. In both the CLN and spleen, there was increased CD8^+^ (*P* = 0.04, *P* = 0.02), Th1 (*P* < 0.0001, *P* = 0.005), and Tbet^+^ CD8^+^ T cells (*P* = 0.002, *P* = 0.002) in males at 14 months compared to 8 months **(**Fig. 2a-d, and 2f**)**.

To help contextualize T cell changes relative to age-matched WT mice, we include average values from 8- and 14-month-old male and female WT animals in Supplementary Table 6. These animals were not part of the current experiment and should not be interpreted as experimental controls; they are provided solely to aid interpretation of the results.

Taken together, these results suggest that males progressively adopt a pro-inflammatory T cell profile with age, while females display a more balanced or regulatory immune environment, highlighting divergent immune aging trajectories between sexes.

## Sex- and age-dependent changes in cytokine-producing T cells

Next, T cells were isolated and stimulated to determine the proportion of interferon gamma (IFNγ), interleukin 17 (IL-17), granulocyte-macrophage colony-stimulating factor (GM-CSF), and interleukin 10 (IL-10) positive T cells. We assessed peripheral T cell cytokine production in the CLN and spleen of male and female 3KL mice at 8 and 14 months of age. We found main effects of both sex and aging across our various cytokine-producing T cells (statistical factors reported in Supplementary Table 7).

**Fig. 3:**
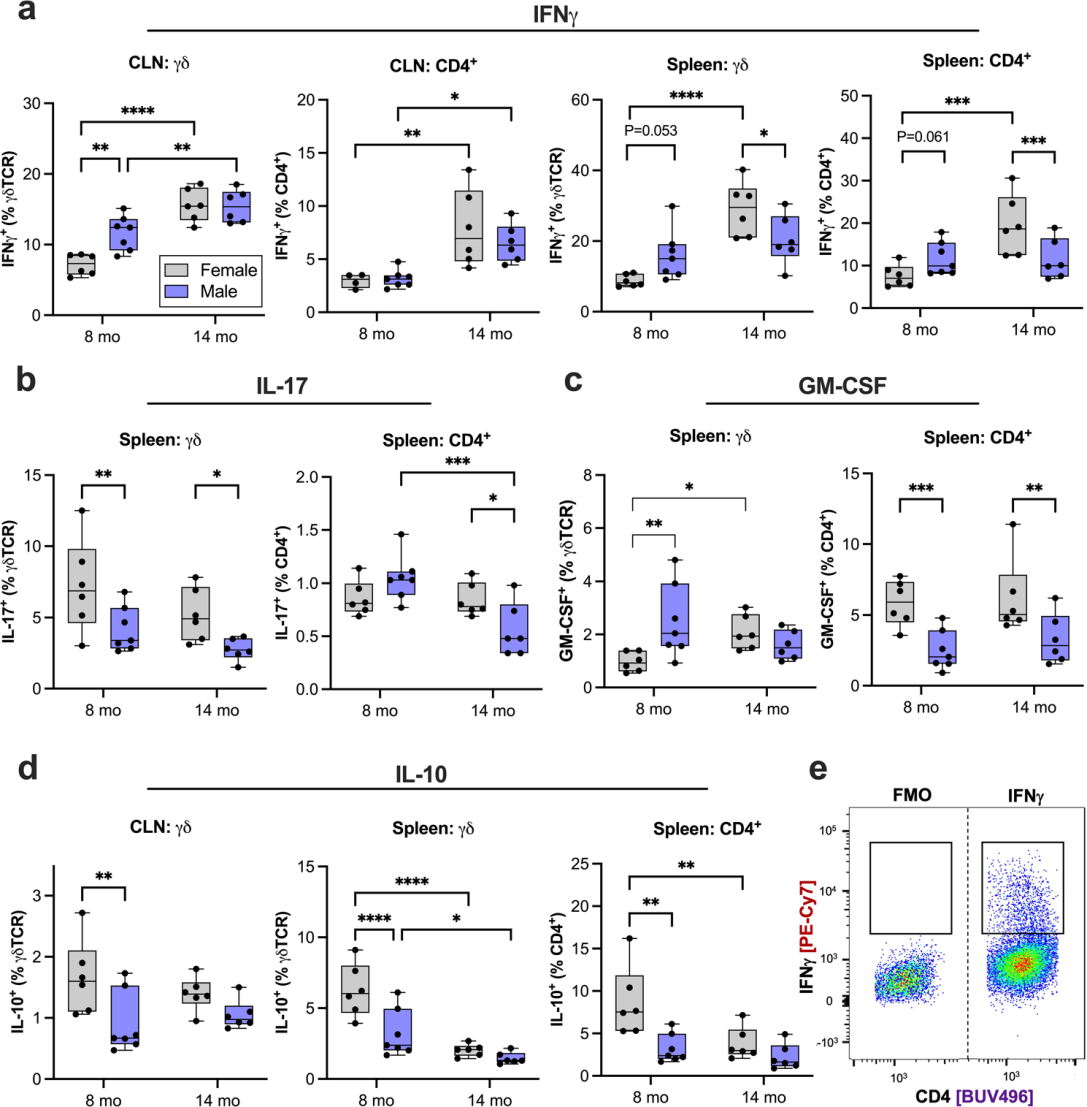
Age and sex-dependent cytokine-producing T cell alterations in 3KL mice. **(a)** IFN-producing, **(b)** IL-17-producing, **(c)** GM-CSF-producing, and **(d)** IL-10-producing and CD4^+^ T cells from cervical lymph nodes and spleen. **(e)** Representative plot IFN fluorescence minus one (FMO) and IFNγ-producing CD4^+^ T cells from cervical lymph node. Whiskers of box and whisker plot represent minimum and maximum. Grey boxes are female data; blue boxes are male data. Data were analyzed by mixed effect analysis with post-hoc Fisher’s LSD. *P* < 0.05 was considered significant. *P < 0.05, **P < 0.01, ***P < 0.001, ***P < 0.0001

Examining early sex-specific differences at 8 months, male 3KL mice had increased IFN-producing T cells in the CLN (*P* = 0.001) **(**Fig. 3a**)**, a trend to increased IFNγ-producing and CD4^+^ T cells in the spleen (*P* = 0.053, *P* = 0.061) **(**Fig. 3a**)** and increased splenic GM-CSF-producing T cells (*P* = 0.009) **(**Fig. 3c**)**. Conversely, female mice had increased IL-17- and IL-10-producing γδ T cells (*P* = 0.009, *P* < 0.0001) **(**Fig. 3b and d**)** and GM-CSF- and IL-10-producing CD4^+^ T cells (*P* = 0.005, *P* = 0.002) **(**Fig. 3c and d**)** in the spleen, and increased IL-10-producing T cells in the CLN (*P* = 0.005) **(**Fig. 3d**)** compared to males.

We also examined age-related immune changes separately in females and males. 14-month-old vs. 8-month old females had increased IFNγ-producing and CD4^+^ T cells in the CLN (*P* = 0.03, *P* = 0.0006) **(**Fig. 3a**)**, IFN-producing CD4^+^ (*P* = 0.0005), GM-CSF-producing (*P* = 0.04) in the spleen **(**Fig. 3a and c**)**, and decreased IL-10-producing splenic CD4^+^ T cells (*P* = 0.003) **(**Fig. 3d**)** compared to 8 months. 14 months old males had increased IFNγ-producing γδ and CD4^+^ T cells in the CLN (*P* < 0.0001, *P* = 0.01) **(**Fig. 3a**)**, and decreased IL-17-producing CD4^+^ T cells in the spleen (*P* = 0.02) compared to 8 months of age **(**Fig. 3b**)**.

To help contextualize cytokine changes relative to age-matched WT mice, we include average values from 8- and 14-month-old male and female WT animals in Supplementary Table 8. These animals were not part of the current experiment and should not be interpreted as experimental controls; they are provided solely to aid interpretation of the results.

In summary, cytokine analysis demonstrates that males are biased toward IFN-driven pro-inflammatory responses, whereas females show early enrichment of IL-17, GM-CSF, and IL-10, consistent with a more mixed but potentially protective immune profile.

### Microglial transcriptional differences are prominent at 8 months and diminish with aging

**Fig. 4:**
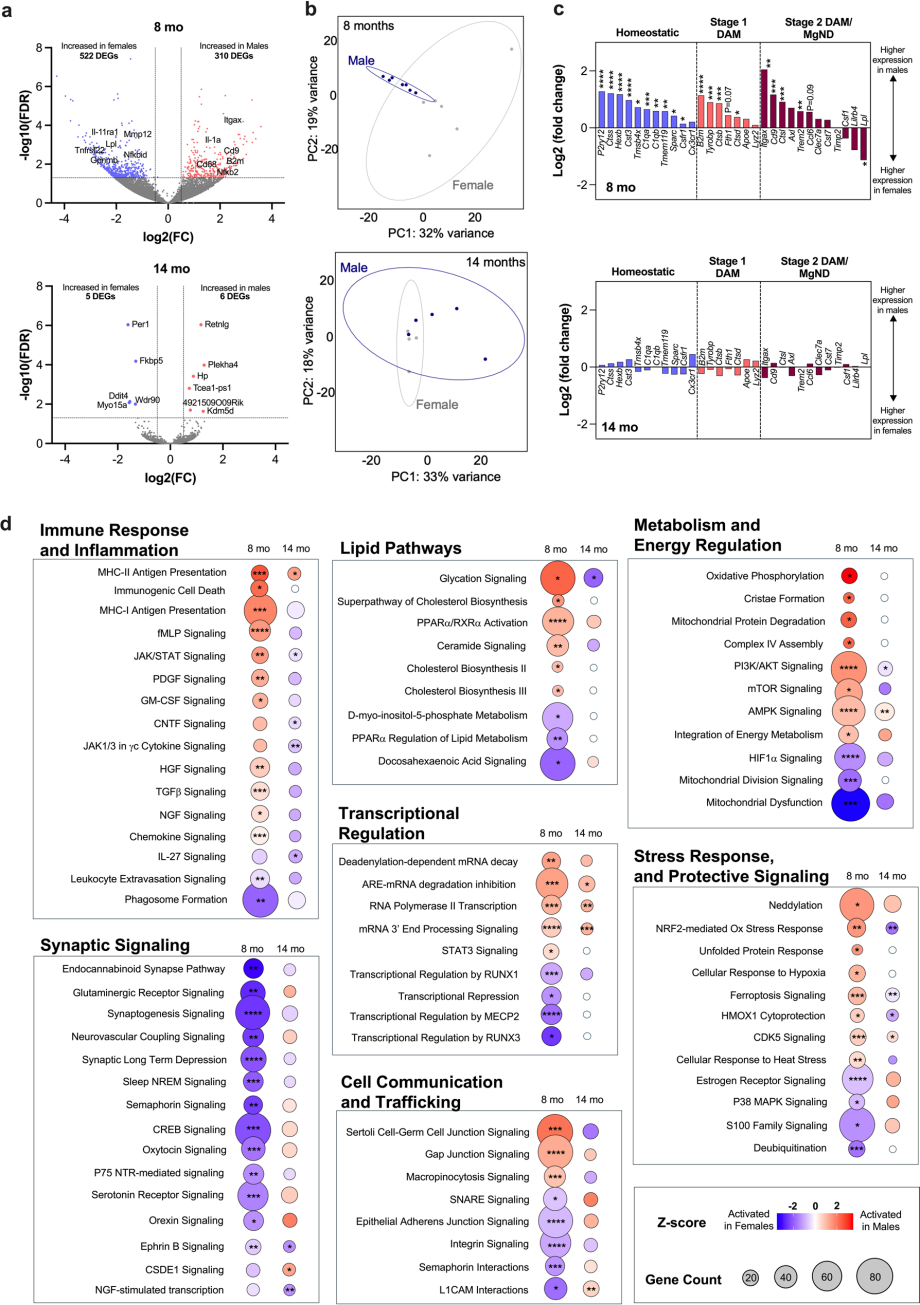
Microglia from male 3KL mice show an early inflammatory profile at 8 months of age. **(a)** Volcano plots of the differentially expressed genes of FACS sorted microglia at 8- and 14-months old. **(b)** Principal coordinate analysis (PCoA) plots showing the variations between male and female microglial transcriptional profiles. **(c)** Gene expression fold change of homeostatic, Stage 1 DAM and Stage 2 DAM microglia. **(d)** Activated pathways from isolated of FACS sorted microglia at 8- and 14-months old determined by Ingenuity Pathway Analysis. Blue represented activation in females compared to males, red represents activation in males compared to females. Differentially expressed gene significant differences detected by DESeq2. *P < 0.05, **P < 0.01, ***P < 0.001, ***P < 0.0001

Microglia from male and female mice were FACS sorted and transcriptional profiles were investigated by RNA sequencing. At 8 months of age, there were 832 differentially expressed genes (FDR q < 0.05), with 522 increased in females and 310 increased in males (Fig. 4a and b**)**. Y-linked genes including *Eif2s3y*,* Uty*,* Ddx3y*, and *Kdm5d* were removed from analyses. With regards to markers of microglial phenotypes, at 8 months male mice had increased expression of markers associated with homeostasis and synaptic pruning [27, 28], including *P2ry12*, *Tmem119*,* Ctss*, *Hexb*,* Cst3*,* Tmsb4x*,* Sparc*,* C1qa*,* and C1qb.* We also found an increase in microglial neurodegenerative disease (MGnD) signatures and stage I disease-associated microglia (DAM) signatures were also increased, with marked expression of *B2m*,* Tyrobp*,* Ctsb*, and *Ctsd* in male mice [29]. Furthermore, Stage II DAM signatures were increased, with elevated expression of *Itgax*,* Cd9*,* Ctsl*, and *Trem2* [29]. Only a single gene, *Lpl* was increased in female mice. Importantly, these changes were no longer significant between males and females at 14 months (Fig. 4c**)** when both sexes show motor deficits and estrogen levels significantly drop in aged female mice.

Next, altered microglial pathways were identified using Ingenuity Pathway Analysis. At 8 months, male microglia showed upregulation of multiple immune-related pathways, including MHC class I and II antigen presentation, granulocyte macrophage colony stimulating factor (GM-CSF) and formyl-methionine-leucine-phenylalanin (fMLP) signaling that is involved in neutrophil degranulation, and immunogenic cell death, suggesting heightened inflammatory and antigen-presenting activity [30, 31]. In contrast, females showed greater activation of phagosome formation and TGF-β signaling, indicating enhanced phagocytic and regulatory capacity [32, 33]. Lipid pathway analysis revealed increased cholesterol biosynthesis, ceramide, and glycation signaling in males, consistent with lipid accumulation and metabolic stress [34, 35]. Females exhibited greater activation of DHA signaling and PPARα-mediated lipid regulation, suggesting enhanced lipid homeostasis [36, 37] (Fig. 4d**)**.

Energy metabolism pathways were also sexually divergent. 8-month males demonstrated higher oxidative phosphorylation, mitochondrial biogenesis, and growth signaling (mTOR, PI3K/AKT), while 8-month females showed greater mitochondrial stress responses, including HIF1α signaling and mitochondrial division [38]. Synaptic signaling pathways, including endocannabinoid, glutamatergic, CREB, and neurovascular coupling, were predominantly elevated in females, suggesting enhanced roles in synaptic support and neuro-glial communication. Males showed increased transcriptional activity, including RNA polymerase II transcription and mRNA stabilization pathways. In contrast, females displayed greater transcriptional repression and epigenetic control through MECP2, RUNX factors, and DNA methylation [39, 40]. Finally, male microglia were enriched for stress response pathways such as NRF2-mediated oxidative stress, unfolded protein response, and ferroptosis signaling. Female microglia favored regulatory pathways including S100 family and estrogen receptor signaling.

Together, these data highlight broad sex-specific transcriptional programming in microglia across immune, metabolic, and neuro-supportive functions at 8 months of age. Surprisingly, we found few differences between males and females at 14 months, suggesting that immunologic pathways converge in late-stage disease.

## Discussion

Our study demonstrates that sex profoundly shapes both behavioral and disease trajectories in the 3KL model of synucleinopathy. Male mice exhibited earlier and more pronounced impairments in motor coordination, endurance, and spatial memory, with deficits becoming apparent by 8 months and worsening with age. We observed that circulating estrogen was elevated in 8-month-old females, coinciding with protection from motor and cognitive impairments. By 14 months, estrogen levels had declined to near-male levels, aligning with the emergence of behavioral deficits. This correlation is further supported by the beneficial effects of estradiol supplementation in 3KL mice and by the growing body of evidence establishing estrogen as a neuroprotective factor in PD [11, 22, 41]. These results replicate previously reported sex differences in motor and cognitive decline in the 3KL mice, confirming that our cohort aligns with established findings [22, 23].

Building on this, our integration of behavioral assessments with peripheral immune and microglial transcriptional profiles reveals coordinated age- and sex-dependent responses that mirror differences in symptom onset and progression. At 8 months, male mice show a pro-inflammatory immune profile marked by reduced regulatory T cells, elevated IFNγ-producing subsets, and microglial activation programs enriched for antigen presentation and lipid dysregulation. In contrast, females at the same age exhibit immune signatures consistent with protection, including increased GM-CSF, IL-17, and IL-10 production alongside microglial pathways supporting phagocytosis, lipid homeostasis, and synaptic regulation. These coordinated responses may contribute to preserved cognition and delayed motor impairment in females, highlighting early divergence in immune–neural interactions that shape subsequent disease course.

We hypothesized that these behavioral phenotypes were linked to dynamic shifts in peripheral T cell populations. Males showed a decline in total helper CD4^+^ T cells and regulatory subsets, coupled with increases in cytotoxic CD8^+^ and IFNγ-producing Th1 cells, suggesting a shift toward a pro-inflammatory immune profile. A reduction of peripheral CD4^+^ T cells has been reported in PD [42], and numerous studies have described the potential contribution of IFNγ in PD-related neurodegeneration [43–45]. Elevated IFN levels have been reported in PD patient plasma, serum and brain [44, 46, 47]. Importantly, in the MPTP neurotoxin model of PD, IFN-deficiency attenuates dopaminergic degeneration by inhibiting microgliosis [44], indicating a direct contribution to PD pathogenesis. We found that 3KL males have an early elevation in IFN, while female mice displayed a delayed immune activation trajectory with increased IFN levels emerging by 14-months.

Female mice have persistently increased proportions of GM-CSF^+^ and IL-17^+^ CD4^+^ and T cells at both 8- and 14-months of age, alongside a notable early elevation in anti-inflammatory IL-10^+^ T cells, which is diminished by 14 months. Sex differences in GM-CSF and IL-17 expression have been observed in response to immunologic challenges, such as those elicited by infection or excess -synuclein. Female resistance to infection with *Leishmania mexicana* has been attributed to increased serum GM-CSF [48], and uropathogenic *E*. *coli* leads to a robust increase in IL-17 expression in female mice, leading to faster resolution of infection [49]. GM-CSF can cross the blood-brain barrier and engage receptors on immune and neural cells [50], affecting neuronal plasticity, learning and memory [51], potentially contributing to the preserved cognitive function in female 3KL mice in this study. Additionally, GM-CSF has been reported as neuroprotective in neurotoxin models of PD, including MPTP [52], 6-hydroxydopamine [53], and paraquat [54]. Additionally, GM-CSF has been shown to protect dopaminergic neurons in the MPTP model by modulation of innate and adaptive immunity including increased Treg regulation of neuroinflammation [55]. Thus, early immunologic elevations of GM-CSF observed in this study may contribute to the relative neuroprotection observed in females.

IL-17 in is thought to be detrimental in PD by activating microglia and contributing to neuroinflammation [56]. Surprisingly, female mice in this study have a higher proportion of IL-17-producing T cells. However, this higher peripheral IL-17 may be counterbalanced by the early elevation of IL-10-producing T regulatory cells that limit inflammation and maintain immune homeostasis. While IL-17 and GM-CSF are often considered detrimental in chronic inflammation, we speculate that elevated levels in females could play a dual role, supporting early immune clearance of misfolded proteins, modulating microglial function, and preventing a more harmful Th1/cytotoxic shift seen in males, thereby potentially contributing to delayed PD onset. Because the majority of PD experimental investigations have utilized male mice [57, 58] this female-specific protective association of elevated IL-17 in the context of elevated IL-10 may have been overlooked.

We also detected sex-specific differences in neuroinflammation in the CNS. Male and female 3KL mice demonstrated distinct microglial transcriptional states at 8 but not 14 months, which aligned with their behavioral, hormonal, and peripheral immune phenotypes. At 8 months of age, male microglia exhibit increased expression of both homeostatic and microglial neurodegenerative disease (MGnD)/disease-associated microglia (DAM) markers, suggesting a complex activation profile. The DAM phenotype has been shown to develop in a stepwise fashion, with Stage 1 DAM microglia retaining elements of the homeostatic gene signature prior to full transition to Stage 2 DAM [29]. This apparent co-expression likely reflects a heterogeneous microglial population in which subsets of cells exist in transitional activation states. Notably, we observed widespread upregulation of genes associated with Stage 1 DAM, including *B2m* and *Tyrobp*, alongside emerging increases in select Stage 2 DAM genes such as *Itgax*,* Cd9* and *Trem2*, indicating that male microglia are beginning to progress along the DAM activation trajectory. Additionally, sex-specific differences in microglial responsiveness and immune priming have been reported in various neurodegenerative contexts and may contribute to the broader transcriptional activity observed in males [59]. Together, these findings suggest that 8-month male microglia occupy an intermediate activation state characterized by early DAM/MGnD engagement and partial retention of homeostatic identity, which may reflect enhanced sensitivity to pathological cues in males vs. females this PD model.

Microglial pathway enrichment further highlighted sex differences, with heightened pro-inflammatory signaling and lipid dysregulation in male microglia and activated synaptic pathways dominating female microglia. There is a strong activation of MHC class I and II antigen presentation in males, suggesting the microglia are in an immunologically active state. In the PD brain, MHC-positive microglia present autoantigens and induce T cell-mediated cytotoxicity [60], consistent with our observed increase in cytotoxic CD8^+^ T cells in males, and the number of MHC class II-positive microglia increases proportionally to the neuronal damage in the *substantia nigra* [61]. Conversely, females have enriched phagosome formation, which indicates enhanced capacity for phagocytosis and debris clearance, which is disrupted in PD [62].

In addition to immune pathways, we also found sex-specific differences in microglial metabolic pathways. Male microglia showed indications of lipid dysregulation, with robust enrichment of cholesterol biosynthesis pathways, as well as ceramide signaling, and glycation-related pathways, indicating a shift toward pro-inflammatory lipid signaling. Ceramide is a bioactive sphingolipid and a positive modulator of NLRP3 inflammasome assembly in microglia [63, 64], and ceramide signaling is linked to chronic inflammation and are dysregulated in PD [65]. Glycation signaling is another key pathway that is activated in the males at 8 months. In vitro studies have shown that glycation accelerates α-synuclein oligomerization [66], and glycated α-synuclein oligomers induced NLRP3 inflammasome-mediated neuroinflammation in microglia [67]. In contrast, female microglia showed enrichment for docosahexaenoic acid signaling, which inhibits release of proinflammatory mediators and enhances phagocytic activity in microglia, hypothesized to limit neuronal damage [68]. Importantly, activation of PPARα, as is seen in the female microglia, reduces amyloid plaque pathology and restores cognition in a mouse model of Alzheimer’s disease [69]. These findings suggest that male and female microglia engage distinct lipid signaling cascades, possibly reflecting differential strategies for responding to neurodegenerative stress. These findings also provide additional insight into reports that decreasing LIPE, a major lipid degrading enzyme, only provided protection in male 3KL mice [70].

In female microglia at 8 months, the upregulated pathways suggest a regulatory and neuroprotective phenotype, marked by enhanced engagement in synaptic signaling and transcriptional regulation. Microglia play a critical role in synaptic development and plasticity [71], and the activation of synaptogenesis signaling at 8 months in females may reflect an effort to maintain synaptic integrity, which is disrupted in PD [72]. Notably, microglia express metabotropic glutamate receptors, and glutaminergic signaling is activated in female microglia. Activation of these receptors has been shown to attenuate α-synuclein-induced inflammation in vitro and confer anti-inflammatory and neuroprotective effects in vivo following LPS challenge [73].

While there were prominent sex-specific microglial changes at 8 months, these differences had disappeared by 14 months of age. Reduction of male and female immunologic changes at 14 months is concurrent with an age-related decline in estrogen observed female 3KL mice. Estrogen is a key regulator of microglial function, and declining estrogen levels have been shown to alter microglial activation states in PD and PD models. Progression of neuroinflammation-mediated neurodegeneration in PD is accelerated in menopausal women [74], and bilateral ovariectomy eliminates female-associated protection against LPS-induced microglial activation and dopaminergic degeneration [75], This suggests that aged females are equally as vulnerable to neuroinflammatory effects of synuclein pathogenesis as males but exhibit a slower disease course that may be mediated by the protective effects of estrogen. Given the increase in the population of aged individuals, this data has important implications for evaluating sex-specific risk to synucleinopathies and suggests different windows of vulnerability to disease onset and potential for of immunologic therapeutic intervention.

Our findings reveal sex-dependent that differences in peripheral immune and neuroimmune landscapes of the 3KL synucleinopathy model that are associated with altered disease severity. More severe motor and cognitive deficits observed in male mice coincided with a pro-inflammatory peripheral immune profile, and microglial signatures, indicative of heightened stress sensitivity, broad DAM activation, and lipid dysregulation. In contrast, female mice show delayed symptom onset, preserved cognitive function, and a peripheral immune environment enriched in GM-CSF and IL-17-producing T cells, which may support regulatory and neuroprotective microglial phenotypes marked by engagement in synaptic signaling and transcriptional plasticity. Importantly, these transcriptional and pathway-level differences were most prominent at 8 months of age, a time point representing early to mid-stage disease, and largely dissipated by 14 months, suggesting a convergence of microglial states across sexes in later disease stages.

These findings are consistent with the seminal work by Rajsombath *et al.*, which characterized the α-synuclein landscape in 3K mice at six months of age [76]. Although total human α-synuclein levels did not differ between males and females, females exhibited a significantly higher tetramer-to-monomer ratio, a feature associated with delayed phenotypic onset. Notably, highly aggregate-prone phosphorylated monomers can activate T-cell responses (reviewed in [77]), and α-synuclein aggregates released from neurons activate microglia [78]. Together, these observations raise the possibility that sex-dependent immune activation in 3K mice is initiated by shifts in the tetramer:monomer ratio, a change that emerges earlier in males and is modulated by estrogen [76]. Heightened immune reactivity to these monomeric, aggregation-prone species may then exacerbate disease progression in males. Conversely, a more restrained immune response in females may allow more effective handling of pathogenic monomers, preserving tetrameric stability and delaying pathology. Further mechanistic investigation into the interplay among phosphorylated -synuclein species, estrogen signaling, and immune pathways will be critical for understanding the inflammatory mechanisms that may contribute sex-specific differences in PD progression.

## Conclusions and limitations

Together, these findings reveal that males and females follow distinct early disease trajectories, males exhibit accelerated motor and cognitive decline alongside a pro-inflammatory immune environment, whereas females maintain preserved behavior in the context of a more regulatory and neuroprotective immune signature. With advancing age and declining estrogen, however, these protective responses in females wane, and by 14 months both sexes show converging motor behavior impairment and overlapping microglial states. This temporal coordination between immune environment and behavioral outcomes suggests that the slower disease course observed in females may be mediated, at least in part, by early protective immune mechanisms.

This study has several limitations. First, while we identify clear sex- and age-dependent associations between behavior, peripheral immune profiles, and microglial transcriptional states, the data do not establish causality. Mechanistic links, such as whether GM-CSF or IL-17 directly mediate neuroprotection in females, or whether IFN drives the accelerated decline in males, remain to be tested. Second, although sex differences are likely influenced by hormonal regulation, particularly estrogen, and potentially by sex-linked genetic programs, these drivers were not directly manipulated here. Third, although our transcriptomic findings align with previously reported signatures in neurodegenerative models [27], replication experiments or orthogonal validation approaches (e.g., RT-qPCR) were not performed and would strengthen confidence in these results. Finally, these findings are specific to the 3KL transgenic model and may not capture the full spectrum of mechanisms underlying human PD and related synucleinopathies. Addressing these limitations through hormone manipulations, immune cell perturbation, and complementary behavioral assays will be important next steps to establish causality and strengthen translational relevance.

### Perspectives and significance

This temporal pattern supports a model in which male and female microglia initially follow distinct activation trajectories, pro-inflammatory and stress-responsive in males versus regulatory and neuroprotective in females, yet ultimately arrive at a common activated state as pathology progresses. Whether these differences are a consequence of reduced toxic α-synuclein burden in the female brain, potentially mediated by estrogen [22], leading to dampened immune activation, or whether the distinct female immune response actively limits α-synuclein accumulation - or both -remains to be determined. These findings may have implications not only for PD but also for related -synucleinopathies such as LBD and PDD, where immune responses and cognitive decline are prominent features and also exhibit known sex differences in prevalence, symptomatology, and progression [79–81]. Together, these results provide mechanistic insight into the sex-specific modulation of neurodegeneration and underscore the importance of incorporating sex as a biological variable in the study and treatment of PD.

## Supplementary Information

Supplementary Material 1.

## Data Availability

The RNA sequencing data generated and analyzed in this study have been deposited in the NCBI Sequence Read Archive: BioProject PRJNA1301412.

## Abbreviations

CLN: Cervical lymph node
DAM: Disease-associated microglia
GM-CSF: Granulocyte-macrophage colony-stimulating factor
IFNγ: Interferon gamma
IL-10: Interleukin-10
IL-17: Interleukin-17
LBD: Lewy body dementia
MgND: Microglia neurodegenerative
MHC: Major histocompatibility complex
PD: Parkinson’s disease
PDD: Parkinson’s disease dementia

## References

[1] Tysnes OB, Storstein A. Epidemiology of parkinson’s disease. J Neural Transm (Vienna). 2017;124(8):901–5. 28150045 10.1007/s00702-017-1686-y

[2] Van Den Eeden SK, Tanner CM, Bernstein AL, Fross RD, Leimpeter A, Bloch DA, et al. Incidence of parkinson’s disease: variation by age, gender, and race/ethnicity. Am J Epidemiol. 2003;157(11):1015–22. 12777365 10.1093/aje/kwg068

[3] Zirra A, Rao SC, Bestwick J, Rajalingam R, Marras C, Blauwendraat C, et al. Gender differences in the prevalence of Parkinson’s Disease. Mov Disord Clin Pract. 2023;10(1):86–93. 36699001 10.1002/mdc3.13584 PMC9847309

[4] Pringsheim T, Jette N, Frolkis A, Steeves TDL. The prevalence of parkinson’s disease: a systematic review and meta-analysis. Mov Disord. 2014;29(13):1583–90. 24976103 10.1002/mds.25945

[5] Haaxma CA, Bloem BR, Borm GF, Oyen WJ, Leenders KL, Eshuis S, et al. Gender differences in Parkinson’s disease. J Neurol Neurosurg Psychiatry. 2007;78(8):819–24. 17098842 10.1136/jnnp.2006.103788 PMC2117736

[6] Santos-García D, Laguna A, Hernández-Vara J, de Deus Fonticoba T, Cores Bartolomé C, Feal Painceiras MJ, et al. Sex differences in motor and non-motor symptoms among Spanish patients with Parkinson’s disease. J Clin Med. 2023;12(4):1329. 10.3390/jcm12041329 36835866 10.3390/jcm12041329 PMC9960095

[7] Cerri S, Mus L, Blandini F. Parkinson’s disease in women and men: what’s the difference? J Parkinsons Dis. 2019;9(3):501–15. 31282427 10.3233/JPD-191683 PMC6700650

[8] Raheel K, Deegan G, Di Giulio I, Cash D, Ilic K, Gnoni V, et al. Sex differences in alpha-synucleinopathies: a systematic review. Front Neurol. 2023;14:1204104. 37545736 10.3389/fneur.2023.1204104 PMC10398394

[9] Saunders-Pullman R, Gordon-Elliott J, Parides M, Fahn S, Saunders HR, Bressman S. The effect of Estrogen replacement on early parkinson’s disease. Neurology. 1999;52(7):1417–21. 10227628 10.1212/wnl.52.7.1417

[10] Vlaar T, Kab S, Schwaab Y, Fréry N, Elbaz A, Moisan F. Association of parkinson’s disease with industry sectors: a French nationwide incidence study. Eur J Epidemiol. 2018;33(11):1101–11. 29730746 10.1007/s10654-018-0399-3

[11] Lee YH, Cha J, Chung SJ, Yoo HS, Sohn YH, Ye BS, et al. Beneficial effect of estrogen on nigrostriatal dopaminergic neurons in drug-naïve postmenopausal parkinson’s disease. Sci Rep. 2019;9(1):10531. 31324895 10.1038/s41598-019-47026-6 PMC6642214

[12] Gardai SJ, Mao W, Schüle B, Babcock M, Schoebel S, Lorenzana C, et al. Elevated alpha-synuclein impairs innate immune cell function and provides a potential peripheral biomarker for Parkinson’s disease. PLoS One. 2013;8(8):e71634. 24058406 10.1371/journal.pone.0071634 PMC3751933

[13] Butkovich LM, Houser MC, Tansey MG. α-Synuclein and noradrenergic modulation of immune cells in parkinson’s disease pathogenesis. Front NeuroSci. 2018;12:626. 30258347 10.3389/fnins.2018.00626 PMC6143806

[14] Sulzer D, Alcalay RN, Garretti F, Cote L, Kanter E, Agin-Liebes J, et al. T cells from patients with parkinson’s disease recognize alpha-synuclein peptides. Nature. 2017;546(7660):656–61. 28636593 10.1038/nature22815 PMC5626019

[15] Lindestam Arlehamn CS, Dhanwani R, Pham J, Kuan R, Frazier A, Rezende Dutra J, et al. alpha-Synuclein-specific T cell reactivity is associated with preclinical and early parkinson’s disease. Nat Commun. 2020;11(1):1875. 32313102 10.1038/s41467-020-15626-w PMC7171193

[16] Xia Y, Zhang G, Kou L, Yin S, Han C, Hu J, et al. Reactive microglia enhance the transmission of exosomal α-synuclein via toll-like receptor 2. Brain. 2021;144(7):2024–37. 33792662 10.1093/brain/awab122

[17] Fellner L, Irschick R, Schanda K, Reindl M, Klimaschewski L, Poewe W, et al. Toll-like receptor 4 is required for α-synuclein dependent activation of microglia and astroglia. Glia. 2013;61(3):349–60. 23108585 10.1002/glia.22437 PMC3568908

[18] Park JY, Paik SR, Jou I, Park SM. Microglial phagocytosis is enhanced by monomeric alpha-synuclein, not aggregated alpha-synuclein: implications for parkinson’s disease. Glia. 2008;56(11):1215–23. 18449945 10.1002/glia.20691

[19] Roodveldt C, Labrador-Garrido A, Gonzalez-Rey E, Fernandez-Montesinos R, Caro M, Lachaud CC, et al. Glial innate immunity generated by non-aggregated alpha-synuclein in mouse: differences between wild-type and Parkinson’s disease-linked mutants. PLoS One. 2010;5(10):e13481. 21048992 10.1371/journal.pone.0013481 PMC2964342

[20] Zarranz JJ, Alegre J, Gómez-Esteban JC, Lezcano E, Ros R, Ampuero I, et al. The new mutation, E46K, of α-synuclein causes parkinson and Lewy body dementia. Ann Neurol. 2004;55(2):164–73. 14755719 10.1002/ana.10795

[21] Nuber S, Rajsombath M, Minakaki G, Winkler J, Müller CP, Ericsson M, et al. Abrogating native α-Synuclein tetramers in mice causes a L-DOPA-responsive motor syndrome closely resembling Parkinson’s disease. Neuron. 2018;100(1):75-90.e5. 30308173 10.1016/j.neuron.2018.09.014 PMC6211795

[22] Rajsombath MM, Nam AY, Ericsson M, Nuber S. Female sex and brain-selective estrogen benefit α-Synuclein tetramerization and the PD-like motor syndrome in 3K transgenic mice. J Neurosci. 2019;39(38):7628–40. 31405930 10.1523/JNEUROSCI.0313-19.2019 PMC6750939

[23] Moors TE, Li S, McCaffery TD, Ho GPH, Bechade PA, Pham LN, et al. Increased palmitoylation improves estrogen receptor alpha-dependent hippocampal synaptic deficits in a mouse model of synucleinopathy. Sci Adv. 2023;9(46):eadj1454. 37976363 10.1126/sciadv.adj1454 PMC10957154

[24] Picelli S, Björklund ÅK, Faridani OR, Sagasser S, Winberg G, Sandberg R. Smart-seq2 for sensitive full-length transcriptome profiling in single cells. Nat Methods. 2013;10(11):1096–8. 24056875 10.1038/nmeth.2639

[25] Love MI, Huber W, Anders S. Moderated estimation of fold change and dispersion for RNA-seq data with DESeq2. Genome Biol. 2014;15(12):550. 25516281 10.1186/s13059-014-0550-8 PMC4302049

[26] Patro R, Duggal G, Love MI, Irizarry RA, Kingsford C. Salmon provides fast and bias-aware quantification of transcript expression. Nat Methods. 2017;14(4):417–9. 28263959 10.1038/nmeth.4197 PMC5600148

[27] Butovsky O, Weiner HL. Microglial signatures and their role in health and disease. Nat Rev Neurosci. 2018;19(10):622–35. 30206328 10.1038/s41583-018-0057-5 PMC7255106

[28] Krasemann S, Madore C, Cialic R, Baufeld C, Calcagno N, El Fatimy R, et al. The TREM2-APOE pathway drives the transcriptional phenotype of dysfunctional microglia in neurodegenerative diseases. Immunity. 2017;47(3):566–e819. 28930663 10.1016/j.immuni.2017.08.008 PMC5719893

[29] Keren-Shaul H, Spinrad A, Weiner A, Matcovitch-Natan O, Dvir-Szternfeld R, Ulland TK, et al. A Unique Microglia Type Associated with Restricting Development of Alzheimer’s Disease. Cell. 2017;169(7):1276-90.e17. 10.1016/j.cell.2017.05.018 28602351

[30] Tuluc F, Garcia A, Bredetean O, Meshki J, Kunapuli SP. Primary granule release from human neutrophils is potentiated by soluble fibrinogen through a mechanism depending on multiple intracellular signaling pathways. Am J Physiol-Cell Physiol. 2004;287(5):C1264-72. 15229106 10.1152/ajpcell.00177.2004

[31] Hamilton JA. GM-CSF in inflammation. J Exp Med. 2020;217(1):e20190945. 10.1084/jem.20190945 31611249 10.1084/jem.20190945 PMC7037240

[32] Spittau B, Dokalis N, Prinz M. The role of TGFβ signaling in microglia maturation and activation. Trends Immunol. 2020;41(9):836–48. 32741652 10.1016/j.it.2020.07.003

[33] Villani A, Benjaminsen J, Moritz C, Henke K, Hartmann J, Norlin N, et al. Clearance by microglia depends on packaging of phagosomes into a unique cellular compartment. Dev Cell. 2019;49(1):77-88.e7. 30880002 10.1016/j.devcel.2019.02.014

[34] Loving BA, Bruce KD. Lipid and lipoprotein metabolism in microglia. Front Physiol. 2020;11:393 32411016 10.3389/fphys.2020.00393 PMC7198855

[35] Videira PAQ, Castro-Caldas M. Linking glycation and glycosylation with inflammation and mitochondrial dysfunction in parkinson’s disease. Front NeuroSci. 2018;12:2018. 10.3389/fnins.2018.00381 PMC5999786 29930494

[36] Layé S, Nadjar A, Joffre C, Bazinet RP. Anti-inflammatory effects of Omega-3 fatty acids in the brain: physiological mechanisms and relevance to pharmacology. Pharmacol Rev. 2018;70(1):12–38. 29217656 10.1124/pr.117.014092

[37] Bougarne N, Weyers B, Desmet SJ, Deckers J, Ray DW, Staels B, et al. Molecular actions of PPAR α in lipid metabolism and inflammation. Endocr Rev. 2018;39(5):760–802. 30020428 10.1210/er.2018-00064

[38] Semenza Gregg L. Hypoxia-inducible factors in physiology and medicine. Cell. 2012;148(3):399–408. 22304911 10.1016/j.cell.2012.01.021 PMC3437543

[39] Nan X, Campoy FJ, Bird A. MeCP2 is a transcriptional repressor with abundant binding sites in genomic chromatin. Cell. 1997;88(4):471–81. 9038338 10.1016/s0092-8674(00)81887-5

[40] Taniuchi I, Littman DR. Epigenetic gene silencing by runx proteins. Oncogene. 2004;23(24):4341–5. 15156191 10.1038/sj.onc.1207671

[41] Shulman LM. Is there a connection between estrogen and Parkinson’s disease? Parkinsonism Relat Disord. 2002;8(5):289–95. 15177058 10.1016/s1353-8020(02)00014-7

[42] Jiang S, Gao H, Luo Q, Wang P, Yang X. The correlation of lymphocyte subsets, natural killer cell, and Parkinson’s disease: a meta-analysis. Neurol Sci. 2017;38(8):1373–80. 28497309 10.1007/s10072-017-2988-4

[43] Mangano EN, Litteljohn D, So R, Nelson E, Peters S, Bethune C, et al. Interferon-γ plays a role in paraquat-induced neurodegeneration involving oxidative and proinflammatory pathways. Neurobiol Aging. 2012;33(7):1411–26. 21482445 10.1016/j.neurobiolaging.2011.02.016

[44] Mount MP, Lira A, Grimes D, Smith PD, Faucher S, Slack R, et al. Involvement of interferon-gamma in microglial-mediated loss of dopaminergic neurons. J Neurosci. 2007;27(12):3328–37. 17376993 10.1523/JNEUROSCI.5321-06.2007 PMC6672486

[45] Litteljohn D, Mangano E, Shukla N, Hayley S. Interferon-gamma deficiency modifies the motor and co-morbid behavioral pathology and neurochemical changes provoked by the pesticide Paraquat. Neuroscience. 2009;164(4):1894–906. 19782123 10.1016/j.neuroscience.2009.09.025

[46] Mogi M, Kondo T, Mizuno Y, Nagatsu T. p53 protein, interferon-gamma, and NF-kappaB levels are elevated in the parkinsonian brain. Neurosci Lett. 2007;414(1):94–7. 17196747 10.1016/j.neulet.2006.12.003

[47] Barcia C, Ros CM, Annese V, Gómez A, Ros-Bernal F, Aguado-Yera D, et al. IFN-γ signaling, with the synergistic contribution of TNF-α, mediates cell specific microglial and astroglial activation in experimental models of parkinson’s disease. Cell Death Dis. 2011;2(4):e142. 21472005 10.1038/cddis.2011.17 PMC3122054

[48] Lezama-Davila CM, Oghumu S, Satoskar AR, Isaac-Marquez AP. Sex-associated susceptibility in humans with chiclero’s ulcer: resistance in females is associated with increased serum-levels of GM-CSF. Scand J Immunol. 2007;65(2):210–1. 17257227 10.1111/j.1365-3083.2006.01887.x

[49] Zychlinsky Scharff A, Rousseau M, Lacerda Mariano L, Canton T, Consiglio CR, Albert ML et al. Sex differences in IL-17 contribute to chronicity in male versus female urinary tract infection. JCI Insight. 2019;5(13):e122998 31145099 10.1172/jci.insight.122998 PMC6629110

[50] McLay RN, Kimura M, Banks WA, Kastin AJ. Granulocyte-macrophage colony-stimulating factor crosses the blood–brain and blood–spinal cord barriers. Brain. 1997;120(Pt 11):2083–91. 9397023 10.1093/brain/120.11.2083

[51] Krieger M, Both M, Kranig SA, Pitzer C, Klugmann M, Vogt G, et al. The hematopoietic cytokine granulocyte-macrophage colony stimulating factor is important for cognitive functions. Sci Rep. 2012;2:697. 23019518 10.1038/srep00697 PMC3458247

[52] Kim NK, Choi BH, Huang X, Snyder BJ, Bukhari S, Kong TH, et al. Granulocyte-macrophage colony-stimulating factor promotes survival of dopaminergic neurons in the 1-methyl-4-phenyl-1,2,3,6-tetrahydropyridine-induced murine Parkinson’s disease model. Eur J Neurosci. 2009;29(5):891–900. 19245369 10.1111/j.1460-9568.2009.06653.x

[53] Choudhury ME, Sugimoto K, Kubo M, Nagai M, Nomoto M, Takahashi H, et al. A cytokine mixture of GM-CSF and IL-3 that induces a neuroprotective phenotype of microglia leading to amelioration of (6-OHDA)-induced Parkinsonism of rats. Brain Behav. 2011;1(1):26–43. 22398979 10.1002/brb3.11 PMC3217672

[54] Mangano EN, Peters S, Litteljohn D, So R, Bethune C, Bobyn J, et al. Granulocyte macrophage-colony stimulating factor protects against substantia nigra dopaminergic cell loss in an environmental toxin model of Parkinson’s disease. Neurobiol Dis. 2011;43(1):99–112. 21377529 10.1016/j.nbd.2011.02.011

[55] Kosloski LM, Kosmacek EA, Olson KE, Mosley RL, Gendelman HE. GM-CSF induces neuroprotective and anti-inflammatory responses in 1-methyl-4-phenyl-1,2,3,6-tetrahydropyridine intoxicated mice. J Neuroimmunol. 2013;265(1–2):1–10. 24210793 10.1016/j.jneuroim.2013.10.009 PMC3872482

[56] Liu Z, Qiu AW, Huang Y, Yang Y, Chen JN, Gu TT, et al. IL-17A exacerbates neuroinflammation and neurodegeneration by activating microglia in rodent models of Parkinson’s disease. Brain Behav Immun. 2019;81:630–45. 31351185 10.1016/j.bbi.2019.07.026

[57] Zeiss CJ, Allore HG, Beck AP. Established patterns of animal study design undermine translation of disease-modifying therapies for Parkinson’s disease. PLoS One. 2017;12(2):e0171790. 28182759 10.1371/journal.pone.0171790 PMC5300282

[58] Silva RH, Lopes-Silva LB, Cunha DG, Becegato M, Ribeiro AM, Santos JR. Animal approaches to studying risk factors for Parkinson’s disease: a narrative review. Brain Sci. 2024;14(2):156. 10.3390/brainsci14020156 38391730 10.3390/brainsci14020156 PMC10887213

[59] Kodama L, Gan L. Do microglial sex differences contribute to sex differences in neurodegenerative diseases? Trends Mol Med. 2019;25(9):741–9. 31171460 10.1016/j.molmed.2019.05.001 PMC7338035

[60] Gu R, Pan J, Awan MUN, Sun X, Yan F, Bai L, et al. The major histocompatibility complex participates in Parkinson’s disease. Pharmacol Res. 2024;203:107168. 38583689 10.1016/j.phrs.2024.107168

[61] Imamura K, Hishikawa N, Sawada M, Nagatsu T, Yoshida M, Hashizume Y. Distribution of major histocompatibility complex class II-positive microglia and cytokine profile of Parkinson’s disease brains. Acta Neuropathol. 2003;106(6):518–26. 14513261 10.1007/s00401-003-0766-2

[62] Tremblay M-E, Cookson MR, Civiero L. Glial phagocytic clearance in Parkinson’s disease. Mol Neurodegener. 2019;14(1):16. 30953527 10.1186/s13024-019-0314-8 PMC6451240

[63] Scheiblich H, Schlütter A, Golenbock DT, Latz E, Martinez-Martinez P, Heneka MT. Activation of the NLRP3 inflammasome in microglia: the role of ceramide. J Neurochem. 2017;143(5):534–50. 28940479 10.1111/jnc.14225

[64] Custodia A, Aramburu-Núñez M, Correa-Paz C, Posado-Fernández A, Gómez-Larrauri A, Castillo J, et al. Ceramide metabolism and Parkinson’s disease-therapeutic targets. Biomolecules. 2021. 10.3390/biom11070945. 34202192 10.3390/biom11070945 PMC8301871

[65] Belarbi K, Cuvelier E, Bonte M-A, Desplanque M, Gressier B, Devos D, et al. Glycosphingolipids and neuroinflammation in Parkinson’s disease. Mol Neurodegener. 2020;15(1):59. 33069254 10.1186/s13024-020-00408-1 PMC7568394

[66] Shaikh S, Nicholson LF. Advanced glycation end products induce in vitro cross-linking of α‐synuclein and accelerate the process of intracellular inclusion body formation. J Neurosci Res. 2008;86(9):2071–82. 18335520 10.1002/jnr.21644

[67] Kumari M, Bisht KS, Ahuja K, Motiani RK, Maiti TK. Glycation produces topologically different α-Synuclein oligomeric strains and modulates microglia response via the NLRP3-Inflammasome pathway. ACS Chem Neurosci. 2024;15(20):3640–3654 10.1021/acschemneuro.4c00057 39320935

[68] Wieczorek-Szukala K, Markiewicz M, Walczewska A, Zgorzynska E. Docosahexaenoic acid (DHA) reduces LPS-induced inflammatory response via ATF3 transcription factor and stimulates Src/Syk signaling-dependent phagocytosis in microglia. Cell Physiol Biochem. 2023;57(6):411–25. 37962278 10.33594/000000668

[69] Luo R, Su L-Y, Li G, Yang J, Liu Q, Yang L-X, et al. Activation of PPARA-mediated autophagy reduces Alzheimer disease-like pathology and cognitive decline in a murine model. Autophagy. 2020;16(1):52–69. 30898012 10.1080/15548627.2019.1596488 PMC6984507

[70] Adom MA, Hahn WN, McCaffery TD, Moors TE, Zhang X, Svenningsson P, et al. Reducing the lipase LIPE in mutant α-synuclein mice improves Parkinson-like deficits and reveals sex differences in fatty acid metabolism. Neurobiol Dis. 2024;199:106593. 38971480 10.1016/j.nbd.2024.106593 PMC11577057

[71] Andoh M, Koyama R. Microglia regulate synaptic development and plasticity. Dev Neurobiol. 2021;81(5):568–90. 33583110 10.1002/dneu.22814 PMC8451802

[72] Soukup SF, Vanhauwaert R, Verstreken P. Parkinson’s disease: convergence on synaptic homeostasis. Embo J. 2018;37(18):e98960 30065071 10.15252/embj.201898960 PMC6138432

[73] Zhang Y-N, Fan J-K, Gu L, Yang H-M, Zhan S-Q, Zhang H. Metabotropic glutamate receptor 5 inhibits α-synuclein-induced microglia inflammation to protect from neurotoxicity in Parkinson’s disease. J Neuroinflammation. 2021;18(1):23. 33461598 10.1186/s12974-021-02079-1 PMC7814625

[74] Usman S, Mondal AC. Menopause triggers microglia-associated neuroinflammation in Parkinson’s disease. Brain Res. 2025;1859:149649. 40250746 10.1016/j.brainres.2025.149649

[75] Wu S-Y, Chen Y-W, Tsai S-F, Wu S-N, Shih Y-H, Jiang-Shieh Y-F, et al. Estrogen ameliorates microglial activation by inhibiting the Kir2.1 inward-rectifier K + channel. Sci Rep. 2016;6(1):22864. 26960267 10.1038/srep22864 PMC4785403

[76] Rajsombath MM, Nam AY, Ericsson M, Nuber S. Female sex and Brain-Selective Estrogen benefit α-Synuclein tetramerization and the PD-like motor syndrome in 3K Transgenic mice. J Neurosci. 2019;39(38):7628. 31405930 10.1523/JNEUROSCI.0313-19.2019 PMC6750939

[77] Garretti F, Monahan C, Sette A, Agalliu D, Sulzer D. T cells, α-synuclein and Parkinson disease. Handb Clin Neurol. 2022;184:439–55. 35034753 10.1016/B978-0-12-819410-2.00023-0 PMC10193709

[78] Wang S, Chu C-H, Stewart T, Ginghina C, Wang Y, Nie H, et al. α-Synuclein,a chemoattractant, directs microglial migration via H < sub > 2 O < sub > 2-dependent Lyn phosphorylation. Proceedings of the National Academy of Sciences. 2015;112(15):E1926-E35. 10.1073/pnas.1417883112 PMC4403145 25825709

[79] Wyman-Chick KA, Ferman TJ, Armstrong MJ, Chrenka EAB, Chiu SY, Patel B, et al. Sex differences in prodromal dementia with Lewy bodies using the National Alzheimer’s Coordinating Center data. Alzheimers Dement. 2025;21(5):e70275. 40390234 10.1002/alz.70275 PMC12089076

[80] Freuchet A, Pinçon A, Sette A, Lindestam Arlehamn CS. Inflammation and heterogeneity in synucleinopathies. Front Immunol. 2024;15:1432342. 39281666 10.3389/fimmu.2024.1432342 PMC11392857

[81] Kouli A, Camacho M, Allinson K, Williams-Gray CH. Neuroinflammation and protein pathology in parkinson’s disease dementia. Acta Neuropathol Commun. 2020;8(1):211. 33272323 10.1186/s40478-020-01083-5 PMC7713145

