# Supplementary material for: Sex-dependent immune activation shapes disease progression in a model of Parkinson’s disease
Source: Biol Sex Differ. 2025 Dec 24;17:13. doi: 10.1186/s13293-025-00809-1 (PMC12849739; doi:10.1186/s13293-025-00809-1)
Supplement: Supplementary file 1 — Supplementary Material 1. [file 13293_2025_809_MOESM1_ESM.docx]

**SUPPLEMENTARY DATA**

**Supplementary Table 1. T Cell flow cytometry antibodies**

| **Target** | **Fluorophore** | **Dilution** | **Company** | **Catalogue** |
| --- | --- | --- | --- | --- |
| TCRβ | APC-Cy7 | 1:800 | BD Pharmingen | 560656 |
| γδ TCR | APC | 1:400 | Invitrogen | 17-5711-82 |
| CD4 | BUV496 | 1:800 | BD Horizon | 612952 |
| CD8 | BV711 | 1:800 | BD Horizon | 563046 |
| RORγt | BV421 | 1:100 | BD Horizon | 562894 |
| FOXP3 | FITC | 1:100 | BD Pharmingen | 560403 |
| Tbet | PE-dazzle | 1:100 | BioLegend | 644828 |

**Supplementary Table 2. Cytokine flow cytometry antibodies**

| **Target** | **Fluorophore** | **Dilution** | **Company** | **Catalogue** |
| --- | --- | --- | --- | --- |
| TCRβ | APC-Cy7 | 1:400 | BD Pharmingen | 560656 |
| γδ TCR | APC | 1:400 | Invitrogen | 17-5711-82 |
| CD4 | BUV496 | 1:800 | BD Horizon | 612952 |
| CD8 | BV785 | 1:800 | BioLegend | 100750 |
| IFNγ | PE-Cy7 | 1:200 | Invitrogen | 25-7311-82 |
| IL-17 | BUV395 | 1:200 | BD Horizon | 585246 |
| IL-10 | PE-dazzle | 1:200 | BioLegend | 505033 |
| GM-CSF | PE | 1:200 | Invitrogen | 12-7331-82 |

**Supplementary Table 3. Antibodies for microglia FACS**

| **Target** | **Fluorophore** | **Dilution** | **Company** | **Catalogue** |
| --- | --- | --- | --- | --- |
| CD45 | FITC | 1:400 | Invitrogen | 2041142 |
| CD11b | PE-Cy7 | 1:200 | Invitrogen | 25-0112-82 |
| ASCA-2 | PE | 1:400 | Miltenyi Biotec | 130-116-244 |
| TCRβ | PerCP | 1:800 | BioLegend | 109228 |
| B220 | PerCP | 1:800 | BD Pharmingen | 553093 |
| NK-1.1 | PerCP | 1:800 | BioLegend | 108726 |
| CD317 | PerCP | 1:800 | BioLegend | 127022 |
| Ly6G | PerCP | 1:800 | BioLegend | 127654 |


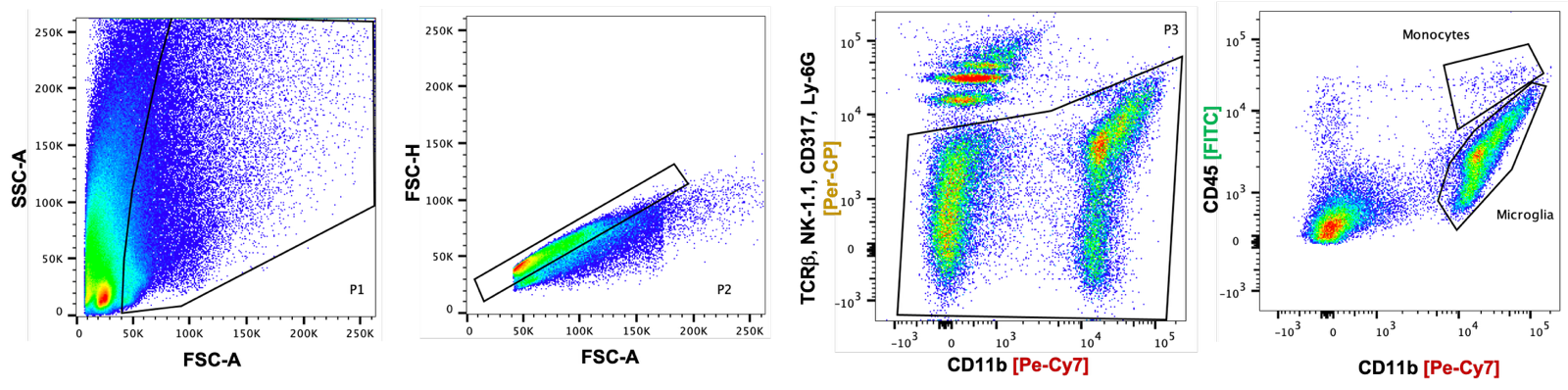


**Supplementary Figure 1.** CD45^int/lo^ CD11b^hi^ microglia gating strategy from homogenized brain samples run on the BDFACSAria^TM^.

**Supplementary Table 4. Reference WT behavior**

| **TEXT FIGURE** | **Test** | **8 months** | | | |
| --- | --- | --- | --- | --- | --- |
|  |  | **F 3KL** | **M 3KL** | **F WT** | **M WT** |
| Fig. 1A | Rota Rod | 221.1±37.8 | 181.1±26.8 | 258.5±29.5 | 222.7±34.7 |
| Fig. 1B | Pole Test | 4.1±0.4 | 5.1±1.3 | 3.8±0.6 | 4.0±0.5 |
| Fig. 1C | Y maze (distance) | 1893.2±197.7 | 1669.9±336.0 | 1952.3±298.0 | 1885.1±321.0 |
| Fig. 1D | Y maze (duration NA) | 79.5±14.5 | 80.0±29.5 | 76.2±11.5 | 85.2±10.9 |
|  |  | **14 months** | | | |
| Fig. 1A | Rota Rod | 219.9±43.5 | 169.3±31.5 | 244.1±22.9 | 209.2±36.1 |
| Fig. 1B | Pole Test | 5.4±0.9 | 6.4±1.6 | 4.1±1.0 | 4.3±1.1 |
| Fig. 1C | Y maze (distance) | 734.5±157.4 | 394.4±114.4 | 1269.4±284.2 | 1163.5±199.7 |
| Fig. 1D | Y maze (duration NA) | 89.9±35.8 | 54.5±27.8 | 69.2±15.2 | 68.2±11.0 |

Data presented as mean ± standard deviation

**Supplementary Table 5. Statistical factors for T cell analysis**

| Fig | Cell | AGE | | SEX | | SEX X AGE | |
| --- | --- | --- | --- | --- | --- | --- | --- |
|  |  | **P value** | **F (DFn, DFd)** | **P value** | **F (DFn, DFd)** | **P value** | **F (DFn, DFd)** |
| 2a | CLN: γδ | 0.495 | F(1,21)=0.48 | 0.6118 | F(1,21)=0.26 | 0.3772 | F(1,21)=0.81 |
|  | CLN: CD4+ | 0.3692 | F(1,11)=0.87 | 0.1308 | F(1,10)=2.70 | **0.0046**** | F(1,11)=13.1 |
|  | CLN: CD8+ | 0.4530 | F(1,11)=0.60 | **0.0455*** | F(1,10)=5.21 | **0.0245*** | F(1,10)=7.00 |
| 2b | Spleen: γδ | **0.0068**** | F(1,11)=11.0 | 0.7214 | F(1,10)=0.13 | 0.4611 | F(1,10)=0.58 |
|  | Spleen: CD4+ | 0.7535 | F(1,21)=0.10 | 0.4151 | F(1,21)=0.69 | **0.0391*** | F(1,21)=4.84 |
|  | Spleen: CD8+ | **0.0145*** | F(1,11)=8.39 | **0.0467*** | F(1,10)=5.14 | 0.5597 | F(1,10)=0.36 |
| 2c | CLN: Treg | **0.0194*** | F(1, 21)=6.41 | **0.0011**** | F(1, 21)=14.2 | 0.6862 | F(1, 21)=0.16 |
|  | CLN: Th17 | **0.0001***** | F(1, 21)=21.3 | **0.0120*** | F(1, 21)=7.56 | **0.0496*** | F(1, 21)=4.34 |
|  | CLN: Th1 | **<0.0001****** | F(1,11)=50.2 | 0.1397 | F(1,10)=2.57 | 0.0754 | F(1,10)=3.93 |
| 2d | Spleen: Treg | **0.0311*** | F(1, 21)=5.33 | 0.3285 | F(1, 21)=1.00 | 0.1654 | F(1, 21)=2.06 |
|  | Spleen: Th17 | **<0.0001****** | F(1,11)=64.6 | 0.0902 | F(1,10)=3.51 | **0.0070**** | F(1,10)=11.4 |
|  | Spleen: Th1 | **0.0050**** | F(1, 21)=9.81 | 0.4892 | F(1, 21)=0.49 | 0.2482 | F(1, 21)=1.41 |
| 2e | CLN: Tbet+ γδ | 0.4817 | F(1, 21)=0.51 | 0.7308 | F(1, 21)=0.12 | **0.0150*** | F(1, 21)=7.01 |
|  | Spleen: Tbet+ γδ | **0.0187*** | F(1, 11)=7.58 | **0.0025**** | F(1,10)=16.1 | 0.4228 | F(1,10)=0.69 |
| 2f | CLN: Tbet+ CD8+ | **0.0007***** | F(1, 21)=15.9 | 0.1064 | F(1, 21)=2.84 | 0.4170 | F(1, 21)=0.68 |
|  | Spleen: Tbet+ CD8+ | **0.0065**** | F(1, 21)=9.11 | 0.1681 | F(1, 21)=2.03 | 0.0549 | F(1, 21)=4.13 |

Mixed effect analysis with post-hoc Fisher’s LSD

**Supplementary Table 6. Reference WT T cell data**

| **CORRESPONDING FIGURE** | **MARKER** | **8 months** | | | |
| --- | --- | --- | --- | --- | --- |
|  |  | **F 3KL** | **M 3KL** | **F WT** | **M WT** |
| Fig. 2A | CLN gd | 1.2±0.2 | 1.1±0.1 | 1.1±0.2 | 1.0±0.2 |
|  | CLN CD4 | 46.2±3.4 | 48.2±1.5 | 48.5±4.1 | 47.5±2.1 |
|  | CLN CD8 | 48.1±3.1 | 47.8±2.5 | 46.4±4.2 | 49.4±2.3 |
| Fig. 2B | Sp gd | 0.7±0.06 | 0.7±0.06 | 0.8±0.1 | 0.5±0.1 |
|  | Sp CD4 | 47.6±9.9 | 55.0±3.1 | 55.6±3.2 | 56.5±3.1 |
|  | Sp CD8 | 31.8±7.5 | 38.3±2.7 | 35.2±3.7 | 37.2±2.8 |
| Fig. 2C | CLN Treg | 20.3±2.5 | 15.4±2.5 | 14.3±2.6 | 18.2±3.3 |
|  | CLN Th17 | 2.0±0.5 | 1.7±0.6 | 2.1±1.1 | 1.2±0.3 |
|  | CLN Th1 | 0.8±0.3 | 0.7±0.4 | 0.8±0.4 | 0.5±0.9 |
| Fig. 2D | Sp Treg | 17.4±1.6 | 15.2±1.7 | 14.1±1.4 | 13.8±1.5 |
|  | Sp Th17 | 2.0±0.4 | 1.5±0.3 | 1.3±0.4 | 0.4±0.1 |
|  | Sp Th1 | 2.6±0.8 | 2.3±0.4 | 1.8±0.3 | 1.5±0.5 |
| Fig. 2E | CLN gd Tbet | 1.3±0.2 | 1.7±0.6 | 0.6±0.3 | 0.1±0.1 |
|  | Sp gd Tbet | 2.6±1.3 | 4.8±1.2 | 3.8±1.3 | 3.5±1.1 |
| Fig. 2F | CLN CD8 Tbet | 1.4±0.4 | 0.8±0.5 | 0.9±0.5 | 0.4±0.5 |
|  | Sp CD8 Tbet | 1.4±0.1 | 1.2±0.3 | 1.0±0.3 | 0.9±0.4 |
|  |  | **14 months** | | | |
| Fig. 2A | CLN gd | 1.0±0.2 | 1.1±0.4 | N/A | 0.9±0.2 |
|  | CLN CD4 | 48.6±5.0 | 42.9±2.5 | N/A | 47.6±2.3 |
|  | CLN CD8 | 46.2±7.0 | 53.0±3.8 | N/A | 47.2±2.6 |
| Fig. 2B | Sp gd | 0.6±0.1 | 0.6±0.1 | 0.9±0.1 | 0.7±0.1 |
|  | Sp CD4 | 53.8±6.7 | 50.4±1.3 | 54.2±3.0 | 55.2±1.4 |
|  | Sp CD8 | 40.0±8.5 | 43.7±1.0 | 36.0±2.2 | 34.6±1.9 |
| Fig. 2C | CLN Treg | 24.6±5.5 | 18.5±3.5 | N/A | 20.0±1.4 |
|  | CLN Th17 | 5.4±2.2 | 3.0±1.0 | N/A | 1.1±0.4 |
|  | CLN Th1 | 2.2±0.9 | 3.2±0.9 | N/A | 0.4±0.3 |
| Fig. 2D | Sp Treg | 14.0±3.5 | 14.4±2.0 | 15.0±1.2 | 16.4±0.9 |
|  | Sp Th17 | 0.6±0.2 | 0.7±0.3 | 1.1±0.2 | 0.4±0.2 |
|  | Sp Th1 | 3.8±1.7 | 5.0±2.6 | 2.0±0.4 | 1.0±0.3 |
| Fig. 2E | CLN gd Tbet | 1.6±0.3 | 1.1±0.4 | N/A | 0.7±0.2 |
|  | Sp gd Tbet | 1.7±0.6 | 3.1±1.4 | 3.3±1.0 | 1.9±0.4 |
| Fig. 2F | CLN CD8 Tbet | 2.0±0.7 | 1.8±0.3 | N/A | 0.3±0.1 |
|  | Sp CD8 Tbet | 1.9±1.2 | 3.6±2.1 | 1.3±0.5 | 1.2±0.1 |

Data presented as mean ± standard deviation

**Supplementary Table 7. Statistical factors for cytokine analysis**

| Fig | Cell | AGE | | SEX | | SEX X AGE | |
| --- | --- | --- | --- | --- | --- | --- | --- |
|  |  | **P value** | **F (DFn, DFd)** | **P value** | **F (DFn, DFd)** | **P value** | **F (DFn, DFd)** |
| 2a | CLN: γδ IFNγ | **<0.0001****** | F(1, 21)=47.9 | **0.0235*** | F(1, 21)=5.96 | **0.0150*** | F(1, 21)=7.02 |
|  | CLN: CD4+ IFNγ | **0.0002***** | F(1, 19)=20.6 | 0.5235 | F(1, 19)=0.42 | 0.3979 | F(1, 19)=0.74 |
|  | Spleen: γδ IFNγ | **<0.0001****** | F(1, 21)=23.0 | 0.7930 | F(1, 21)=0.07 | **0.0053**** | F(1, 21)=9.68 |
|  | Spleen: CD4+ IFNγ | **0.0464*** | F(1, 11)=5.03 | 0.0691 | F(1, 10)=4.14 | **0.0005***** | F(1, 10)=24.9 |
| 2b | Spleen: γδ IL-17 | 0.0868 | F(1, 11)=3.53 | **0.0024**** | F(1, 10)=16.1 | 0.6355 | F(1, 10)=0.23 |
|  | Spleen CD4+ IL-17 | **0.0051**** | F(1, 21)=9.75 | 0.5686 | F(1, 21)=0.33 | **0.0075**** | F(1, 21)=8.75 |
| 2c | Spleen: γδ GM-CSF | 0.7870 | F(1,11)=0.07 | 0.1598 | F(1,10)=2.30 | **0.0149*** | F(1,11)=8.61 |
|  | Spleen: CD4+ GM-CSF | 0.6110 | F(1,11)=0.27 | **<0.0001***** | F(1,10)=41.6 | 0.5380 | F(1,10)=0.40 |
| 2d | CLN: γδ IL-10 | 0.7177 | F(1, 21)=0.13 | **0.0049**** | F(1, 21)=9.87 | 0.2515 | F(1, 21)=1.39 |
|  | Spleen: γδ IL-10 | **0.0010**** | F(1, 11)=19.4 | **<0.0001****** | F(1,10)=62.8 | **0.0002***** | F(1,10)=31.3 |
|  | Spleen: CD4+ IL-10 | **0.0168*** | F(1, 11)=7.93 | **0.0040**** | F(1,10)=13.8 | 0.0638 | F(1,10)=4.34 |

Mixed effect analysis with post-hoc Fisher’s LSD

**Supplementary Table 8. Reference WT cytokine data**

| **CORRESPONDING FIGURE** | **MARKER** | **8 months** | | | |
| --- | --- | --- | --- | --- | --- |
|  |  | **F 3KL** | **M 3KL** | **F WT** | **M WT** |
| Fig. 3A | CLN γδ IFNγ | 7.2±1.5 | 11.6±2.4 | 8.0±2.9 | 8.2±2.8 |
|  | CLN CD4 IFNγ | 3.0±0.7 | 3.2±0.8 | 3.2±1.1 | 3.7±1.1 |
|  | Sp γδ IFNγ | 8.8±1.7 | 16.1±7.1 | 1.5±0.8 | 3.2±1.2 |
|  | Sp CD4 IFNγ | 7.6±2.6 | 11.4±3.9 | 7.2±2.6 | 8.4±3.9 |
| Fig. 3B | Sp γδ IL-17 | 7.2±3.3 | 4.2±1.6 | 6.0±2.8 | 6.1±1.9 |
|  | Sp CD4 IL-17 | 0.9±0.2 | 1.1±0.2 | 0.4±0.1 | 0.7±0.2 |
| Fig. 3C | Sp γδ GM-CSF | 1.4±0.4 | 2.5±1.4 | 1.5±0.8 | 3.7±1.3 |
|  | Sp CD4 GM-CSF | 5.9±1.6 | 2.5±1.4 | 0.8±0.01 | 2.4±1.0 |
| Fig. 3D | CLN γδ IL-10 | 1.7±0.6 | 0.9±0.5 | 1.2±0.5 | 1.1±0.4 |
|  | Sp γδ IL-10 | 6.3±1.9 | 3.2±1.7 | 3.4±1.1 | 1.3±0.5 |
|  | Sp CD4 IL-10 | 8.7±4.1 | 3.2±1.7 | 1.1±0.6 | 0.2±0.1 |
|  |  | **14 months** | | | |
| Fig. 3A | CLN γδ IFNγ | 15.6±2.4 | 15.4±2.3 | N/A | 11.6±3.5 |
|  | CLN CD4 IFNγ | 7.9±3.6 | 6.5±1.8 | N/A | 4.4±0.7 |
|  | Sp γδ IFNγ | 29.1±7.7 | 20.4±7.1 | 9.1±2.2 | 13.0±3.7 |
|  | Sp CD4 IFNγ | 19.6±7.1 | 11.5±4.7 | 8.8±2.6 | 7.8±0.9 |
| Fig. 3B | Sp γδ IL-17 | 5.2±1.9 | 2.8±0.8 | 6.5±0.9 | 7.8±2.5 |
|  | Sp CD4 IL-17 | 0.8±0.2 | 0.6±0.3 | 0.8±0.1 | 0.6±0.2 |
| Fig. 3C | Sp γδ GM-CSF | 2.1±0.6 | 1.6±0.6 | 1.1±0.6 | 2.5±0.2 |
|  | Sp CD4 GM-CSF | 6.2±2.7 | 3.3±1.8 | 1.3±0.7 | 1.5±0.06 |
| Fig. 3D | CLN γδ IL-10 | 1.4±0.3 | 1.1±0.2 | N/A | 1.7±0.6 |
|  | Sp γδ IL-10 | 2.0±0.4 | 1.5±0.4 | 4.0±1.9 | 3.8±0.5 |
|  | Sp CD4 IL-10 | 3.8±1.9 | 2.2±1.5 | 1.3±0.2 | 0.8±0.1 |

Data presented as mean ± standard deviation
